# Comparative *in vitro* safety assessments of synthetized and plant-derived cannabidiol (CBD), and pharmacokinetics of synthetized CBD: evidence of similar toxicological profiles and accumulation upon repeated oral administration

**DOI:** 10.3389/ftox.2026.1844643

**Published:** 2026-09-04

**Authors:** E. T. Wong, D. Bovard, W. K. Schlage, W. Xia, H. Isa, O. Sirbu, A. R. Kolli, G. N. Nikolajsen, S. S. Jensen, J. Ho, B. Phillips, J. Hoeng

**Affiliations:** 1 Verdeya Research Laboratories Pte. Ltd., Singapore, Singapore; 2 Consultant in Biology, Bergisch Gladbach, Germany; 3 Philip Morris Products S.A., Lausanne, Switzerland; 4 Fertin Pharma, Vejle, Denmark; 5 Verdeya SA, Cheseaux sur Lausanne, Lausanne, Switzerland

**Keywords:** cannabidiol, genotoxicity, hepatotoxicity, mutagenicity, repeated oral dosing, pharmacokinetics

## Abstract

Comparative toxicology between synthetized and plant-derived cannabidiol (CBD) remains underexplored, as most previous research has focused on a single type of CBD. This leaves uncertainties regarding whether differences in origin, purity, or extraction methods affect the safety profiles or metabolic outcomes of CBD. This study assessed the *in vitro* safety, *in vitro* hepatotoxicity, and repeated oral dose pharmacokinetics (PK) of CBD in rats. Two brands of synthetized CBD were evaluated alongside two plant-derived CBD (a high-purity isolate and a lower-purity distillate) in vitro. These findings indicated no meaningful in vitro safety differences among the CBD sources and therefore supported selection of a single synthetized CBD source for an in vivo PK study in male Sprague-Dawley rats dosed orally once daily at 26.5 mg/kg for 21 days. Blood samples were collected at multiple time points on days 1 and 21, and pre-dose samples on selected days to assess baseline accumulation. CBD and its metabolites (7-COOH-CBD, 7-OH-CBD, 6-OH-CBD) were measured in plasma and tissues using liquid chromatography-tandem mass spectrometry. All CBD samples showed comparable safety profiles, with no evidence of genotoxicity or mutagenicity in micronucleus and Ames assays, similar cytotoxicity in HepaRG monolayer cultures, and comparable cytochrome P450 (CYP) inhibition. Over 21 days, repeated dosing increased plasma CBD (AUC_INF__D 567 vs. 209 h*kg*ng/mL/mg; Day 21 vs. 1), 7-COOH-CBD (AUC_last__D 1006 vs. 581), 6-OH-CBD (AUC_last__D 33 vs. 13) and 7-OH-CBD (AUC_last__D 43 vs. 31) exposure, and prolonged CBD half-life (41.9 h vs. 5.9 h). The steady-state plasma CBD concentration was achieved by Day 6. Tissue analysis showed marked CBD accumulation in adipose tissue (70,124 ng/g, 1470-fold relative to plasma level) and mesenteric lymph nodes (10,018 ng/g, 201-fold relative to plasma level). Notably, 7-OH-CBD accumulated in adipose tissue (42 ng/g, 21-fold relative to plasma level) and mesenteric lymph nodes (136 ng/g, 73-fold relative to plasma level). Overall, high-purity synthetized and plant-derived CBD showed similar *in vitro* safety outcomes, while oral administration produced distinct accumulation of CBD and metabolites in plasma and tissue. These findings support flexible CBD source selection for standardized nonclinical research while highlighting the need to consider accumulation dynamics during repeated dosing.

## 1 Introduction

The use of purified cannabinoids and extracts derived from *Cannabis sativa L*. for medical or other non-recreational applications has steadily increased over the last years. However, despite this, there remains limited scientific evidence regarding their safety for human consumption. Synthetized CBD needs to be clearly distinguished from synthetic cannabinoids, which are potent analogues of the active components of cannabis that can trigger severe adverse effects across multiple organ systems (Alzu’bi et al., 2024). The European Food Safety Authority (EFSA) has issued a negative opinion on the use of synthetic CBD in food supplement pointing to data gap (the identity, the production process, the compositional data, the specifications, the genotoxicity, the reproductive and developmental toxicity and the human clinical data) identified and hence safety cannot be established (EFSA, 2025). On the other hand, the Advisory Committee on Novel Foods & Processes (ACNFP) assessed a synthetized CBD (>98% pure) dossier for novel food, provisional acceptable daily intake (ADI) of 10 mg/day for a 70 kg healthy adult is recommended, subject to the existing advice to consumers that pregnant and breastfeeding women and people taking any prescription medication should avoid the consumption of CBD (ACNFP, 2025). With the legalization of hemp for medicinal use, a wider range of hemp-derived products has become available. However, the quality of these CBD products can vary significantly, as they may differ in their actual chemical content compared to the product labels as well as being contaminated with residual solvents, heavy metals, pesticides, or microbial pathogens (Montoya et al., 2020; Simei et al., 2024).

*Cannabis sativa* contains over 560 identified and/or isolated compounds including 125 cannabinoids (Morales et al., 2017; Radwan et al., 2021; Fordjour et al., 2023). Among these, CBD stands out as a non-intoxicating compound that has attracted significant scientific and medical interest. Its prominence is underscored by regulatory approval as a treatment for epilepsy (Franco et al., 2021), as well as a growing body of research supporting its potential therapeutic values in managing chronic pain, muscle spasticity, anxiety, psychosis, and substance-abuse disorders (Hoch et al., 2025; Stains et al., 2025).

It is therefore not surprising that the global CBD market size has been estimated at USD 9.14 billion in 2024 and is projected to reach USD 22.05 billion by 2030 (Grand View Research, 2025). Increasing therapeutic use and global commercialization resulted in greater emphasis on the quality, purity, standardization and sourcing of CBD to ensure consistent efficacy, patients’ safety, and regulatory compliance. Pharmaceutical-grade CBD, manufactured under Good Manufacturing Practice (GMP) conditions, is typically defined as containing >98% CBD on dry weight basis, with tightly controlled limits for related substances, including low levels of Δ^9^-tetrahydrocannabinol (THC). Purified CBD from *Cannabis sativa L.* intended for medical use is regulated and standardized through pharmacopeial framework including the European Pharmacopoeia (Ph Eur, 2024) and the proposed (draft) United States Pharmacopoeia (USP) monograph (USP, 2024). Highly purified distillates (∼80% of CBD) can be also used as active pharmaceutical ingredients (APIs) when produced under the GMP conditions, having established stability data and are tested for purity and impurities profile in accordance with the supplier’s specifications and relevant industry standards. Due to different impurity profiles, there is currently no pharmacopeial framework for synthetized CBD.

CBD can be obtained from natural, chemically synthetized or biosynthetic sources, each with advantages and regulatory implications.

1. Hemp-derived CBD is extracted, purified and then isolated from high-CBD, hemp varieties, of *Cannabis sativa L.* CBD from this process is naturally derived and widely accepted in current markets. It consists of highly purified (−)-*trans* CBD and small fractions of minor cannabinoids (e.g., Tetrahydrocannabinol (THC), Cannabidiorcol (CBD-C1), Cannabidivarin (CBDV), Cannabidibutol (CBD-C4)). Surprisingly, the plant-origin CBD preparations containing these other cannabinoids showed improved therapeutic efficacy compared to synthetized CBD which comprises no other cannabinoid constituents (Kogan et al., 2019).
2. Synthetized CBD is produced via stereo-specific expression systems, chemically it is identical to plant-derived CBD, however it will have different impurities depending on the synthesis pathway. The process is consistent and scalable, potentially less costly; however, to clearly exclude the presence of uncontrolled “synthetic cannabinoids” (Alzu’bi et al., 2024), it will come under regulatory scrutiny due to requirements to thoroughly characterize its identity, production process, compositional data and specifications (EFSA, 2022) and no coverage by the Ph. Eur. and USP standards (Alzu’bi et al., 2024).
3. Biosynthetic CBD is produced using genetically engineered microorganisms (e.g., yeast) and is still under development and has limited commercial availability.

This diversity in source materials, quality standards and processing methods results in a broad spectrum of CBD isolates and distillates with differing purity levels and impurity profiles, each of which may influence the safety and efficacy of the final CBD formulation. Although the understanding of CBD safety has advanced significantly in recent years due to a growing body of science-backed data, it remains difficult to generalize these findings across the wide variety of commercial and experimentally available CBD products. This challenge reflects substantial variations in plant source and strain, extraction and purification methods, the composition of cannabinoids and non-cannabinoid constituents, and the experimental designs used in safety assessments. Consequently, differences in product composition limit cross-study comparability and complicate the extrapolation of existing toxicological findings to all CBD formulations. We hypothesize that differences in source materials, manufacturing processes, GMP status, purity, and impurity profile may influence the *in vitro* toxicological profiles of the CBD APIs. Therefore, a head-to-head comparative assessment was required to determine whether such compositional differences translate into API-dependent differences in genotoxicity, cytotoxicity, hepatic functional endpoints, or CYP activity. If no meaningful *in vitro* differences were observed, one well-characterized GMP-grade synthesized CBD API could be selected as a representative material for repeated-dose PK evaluation to contextualize systemic exposure, accumulation, and tissue distribution.

There are many studies evaluating the non-clinical *in vitro* safety of CBD, starting with mutagenicity (Ames assay) and genotoxicity (micronucleus assay), often conducted as US FDA GLP-compliant studies, typically using hemp-derived CBD isolate (>97% purity) (CDER Food and Drug Administration, 2018; Henderson et al., 2023b; Tallon et al., 2025). Using the Ames mutagenicity assay in a standard panel of *S. typhimurium* and *E. coli* bacterial strains, independent groups have assessed a CBD isolate, >99% purity up to 5,000 ug/plate (Henderson et al., 2023b) or a synthetized form of CBD, >97% purity tested up to 15,000 ug/plate (Tallon et al., 2025). In both cases, the results were negative for mutagenicity across the concentration range. Similarly, both groups assessed *in vitro* genotoxicity of CBD (isolate or synthetized) with the micronucleus assay conducted in human lymphoblastoid TK6 cells. In this series of studies, assessments up to 60 μg/mL were negative for genotoxicity in the concentration range within acceptable viability ranges. These results collectively are aligned with the results from the GW Pharma New Drug Application for CBD (Epidiolex) with the FDA (CDER Food and Drug Administration, 2018), indicating no genotoxicity risk in its CBD formulation. However, some data present in the literature have provided alternative outcomes. For example, in Kolar et al. (Kolar et al., 2024), where CBD (and several minor cannabinoids) exhibited a mild but significantly increased micronuclei count following CBD treatment (98.7% purity). This introduces the caveat that the consolidated evaluation of the totality of the genotoxicity data must consider multiple variabilities, including CBD source, purity and impurities profile, excipients or solvents used, concentration range tested, and cell systems used.

In the context of comparative *in vitro* toxicology, PK evaluation is essential to determine whether concentrations associated with observed *in vitro* effects are achievable under physiological conditions and whether repeated dosing may result in accumulation that could influence risk interpretation. Given the variability in published *in vitro* findings and the need to establish their *in vivo* relevance, PK evaluation was undertaken to determine whether systemic exposure, CBD accumulation, or tissue distribution under physiological dosing conditions could influence the translation of these observations. CBD is characterized by low oral bioavailability, attributable to its lipophilic properties and extensive first-pass metabolism (Millar et al., 2020). Most CBD PK studies have focused on the administration of a single oral dose under fasting conditions (O'Sullivan et al., 2024). Notably, co-administration with a high-fat meal has been shown to enhance CBD bioavailability in humans compared to dosing in the fasted state (Taylor et al., 2018; Perucca and Bialer, 2020; Bergeria et al., 2022; Jang et al., 2024; Saals et al., 2025). Repeated administration of CBD to rats has also been associated with dose-dependent accumulation (Taylor et al., 2018; Schwotzer et al., 2025; Xia et al., 2025). Despite these findings, data on the accumulation of CBD and its metabolites, the trough concentrations during repeated oral dosing, and the tissue distribution that occurs following repeated oral administration remain limited.

Accordingly, this study aimed to conduct a head-to-head comparison of four commercial purified (−)-*trans*-CBD APIs (Table 1), including both synthetized and plant-derived materials of varying purity (≥84.3%) and GMP status, through *in vitro* comparative safety assessment. These assessments were designed as a comparative toxicology assessment using standard regulatory assays, providing insight into the consistency of CBD safety profiles across materials differing in origin, purity, and manufacturing. Based on the outcome of the *in vitro* assessment, a single CBD source was then selected for *in vivo* PK evaluation following repeated oral administration to contextualize the *in vitro* findings in terms of systemic exposure and accumulation. Together, this integrated approach enables both comparative assessment of CBD APIs and support contextual interpretation of toxicity findings under physiologically relevant exposure conditions. Importantly, this first head-to-head CBD comparison study provides new evidence that synthetized and plant-derived (−)-*trans*-CBD, regardless of their GMP status or purity (≥84.3%), exhibit no significant differences *in vitro* in terms of genotoxicity, mutagenicity, and hepatotoxicity. Notably, longer CBD half-life and significant accumulation of CBD and its metabolites were observed upon repeated oral administration in rats. Additionally, the achievement of a steady-state trough concentration of CBD in plasma after 6 days of repeated dosing offers valuable new insight into the PK behavior of CBD upon repeated dosing. Collectively, the study established that the intrinsic *in vitro* safety profiles of high purity (≥84.3%) CBD are consistent across synthetized and plant-derived sources, regardless of GMP status. Moreover, the data supports the extrapolation of preclinical PK results to humans, which is a critical advancement for regulatory assessments and risk evaluation of CBD products from diverse origins.

**TABLE 1:** API sources and properties.

CBD used in study	API 1	API 2	API 3	API 4
Source	Non-hemp	Non-hemp	Hemp-derived	Hemp-derived
Type	Synthetized	Synthetized	Isolate	Distillate
Stereoisomer	(−)-*trans* isomer	(−)-*trans* isomer	(−)-*trans* isomer	(−)-*trans* isomer
CBD % (w/w)	99.4	99.5	99.3	84.3
Quality standard in manufacturing	Non-GMP	GMP	GMP	GMP compliant
Characteristics of API	No detected D9-THC (<0.03% w/w)	No detected D9-THC (<0.03% w/w)	Detected total THC (D9-THC, D8-THC, THCA, THCVA, and THCV) < 0.70% (w/w)	Detected total THC (D9-THC, D8-THC, THCA, THCVA, and THCV) < 0.354% (w/w)
Testing strategy (*in vitro*)	*In vitro* micronucleus assay, Ames test, *in vitro* liver cytotoxicity, *in vitro* CYP activity			
Testing strategy (*in vivo*)	Nil	Repeated oral dosing and PK study	Nil	Nil

CBD, cannabidiol; THC, tetrahydrocannabinol; THCA, tetrahydrocannabinolic acid; THCV, tetrahydrocannabivarin; THCVA, tetrahydrocannabivarinic acid.

## 2 Materials and methods

### 2.1 Sources and purity of CBD

The four different APIs used in the study as well as an overview of the assessment schemes are presented in Table 1. The 4 APIs were selected to include synthetized (API 1 and 2) and hemp-derived CBDs (API 3 and 4), non-GMP (API 1 and 4) or GMP compliant (API 2 and 3) sources, as well as purity ranging from 84.3% for distillate to ≥99% for synthetized CBD or isolate. The *in vitro* genotoxicity and toxicity assays were performed using all 4 APIs, while only the synthetized GMP-grade CBD (API2) was used in the *in vivo* rat PK study. For the PK investigation, synthetized GMP-grade CBD (API2) was chosen as representative of the four test items because it has the highest purity, while the *in vitro* safety parameters of all these APIs were comparable and the *in vivo* PK parameters were similar to hemp-derived CBD isolate (Xia et al., 2025).

### 2.2 *In vitro* micronucleus assay

The *in vitro* micronucleus assay was conducted according to OECD TG 487 (OECD, 2016) using Chinese Hamster Ovary (CHO-WBL) cells (Merck, Darmstadt, Germany). The 4 APIs were dissolved in dimethyl sulfoxide (DMSO; Merck) and tested at up to ten concentrations (with a maximum of 1% DMSO in medium), selected based on preliminary dose-range finding and solubility/cytotoxicity limits. Each concentration was assessed in triplicate under three conditions: 4-h exposure with and without metabolic activation (S9 mix from phenobarbital/benzoflavone-induced rat liver, ref. 11–105, Moltox, Boone, NC, USA), and 24-h exposure without metabolic activation. Positive controls (Colchicine, ref. C3915, Merck; Methyl methanesulfonate, ref. 129,925, Merck; Cyclophosphamide monohydrate, ref. C0768, Merck) and solvent controls were included in each experiment.

Cells were seeded in 96-well plates, treated, and then processed using the MicroFlow® kit (Ref. MicroFlow In vitro-250/50 Kit, Litron Laboratories, Rochester, NY, USA) for flow cytometric analysis. This involved sequential staining to distinguish viable nuclei and micronuclei, with at least 5,000 nuclei analyzed per well. Cytotoxicity was determined by relative population doubling (RPD), with the highest concentration targeting 55% ± 5% cytotoxicity (RPD ≥ 40%).

Each test run was deemed acceptable if the positive controls demonstrated a statistically significant increase in the percentage of micronucleus-positive cells (%MN) and if the %MN value for the solvent control fell within the established historical control range (data not shown).

A test item was classified as genotoxic under the following criteria: if the %MN for a specific dose was significantly greater than that observed for the solvent control, if the %MN exceeded the historical control range for the solvent, and if there was a dose-dependent increase in %MN.

All procedures followed validated internal work instructions and were conducted in compliance with Good Laboratory Practice (GLP).

### 2.3 *In vitro* ames assay

The mutagenic potential of the APIs was assessed using the bacterial reverse mutation assay (Ames test) in accordance with OECD Guideline 471 (OECD, 2020), ICH S2 (R1) (ICH, 2012), and validated internal procedures. Five *Salmonella typhimurium* strains (TA98, TA100, TA102, TA1535, TA1537; Covance Laboratories Limited, Harrogate, United Kingdom), each carrying specific mutations to detect base-pair substitutions or frameshift mutations, were employed.

The APIs were dissolved in DMSO and tested at up to six concentrations, selected based on preliminary dose-range finding and solubility/cytotoxicity limits. Each concentration was tested in triplicate, both in the absence and presence of metabolic activation. Positive (4-Nitro-o-phenylenediamine, ref. 108,898, Merck; Sodium azide, ref. 60–120, Moltox, Boone, NC, USA; Mitomycin C, ref. 60–100.11, Moltox; 9-Aminoacridine HCl, ref. 60–147, Moltox; Benzo(a)pyrene, ref. 60–114, Moltox; 2-Aminoanthracene, ref. 60–157.2, Moltox) and solvent controls were included for each strain and condition.

For each assay, overnight bacterial cultures were prepared and confirmed for viability (≥5 × 10^8 CFU/mL). The 4 APIs, S9 mix (for + S9), and bacteria were pre-incubated, then mixed with top agar containing trace histidine/biotin and poured onto minimal glucose agar plates (Ref. 21-40S29, Moltox). Plates were incubated at 37 °C ± 2 °C for 48–72 h. Revertant colonies were counted using an automatic colony counter (Sorcerer Colony Counter, Instem, Staffordshire, United Kingdom) or manually if necessary. Cytotoxicity was assessed by examining the bacterial lawn. Acceptance criteria required spontaneous revertant growth in negative and solvent controls, positive control responses within expected ranges, and sterility of the APIs.

A test substance was classified as mutagenic if any of the following criteria were met: (1) the revertant colony count for a specific dose exceeded the historical range observed for the solvent-treated control; (2) the ratio of revertant colonies at a given dose compared to the solvent control was greater than 2-fold for strains TA98, TA100, and TA102, or greater than 3-fold for strains TA1535 and TA1537; or (3) there was a clear dose-dependent increase in revertant colonies.

All procedures, including preparation of reagents, bacterial stocks, and waste handling, followed validated internal work instructions and quality management protocols. The study was conducted in compliance with Good Laboratory Practice (GLP) principles.

### 2.4 *In vitro* liver cytotoxicity assay

Toxicity and functional effects of the APIs were evaluated using the Nospin HepaRG™ (Ref. NSHPRG; Lonza, Basel, Switzerland). Cells were thawed and seeded in 96-well plates (∼65,000–70,000 cells/well) using William’s E medium (Ref. A1217601, ThermoFisher, Waltham, MA, USA) supplemented with HepaRG™ Thaw, Plate & General Purpose supplement (ref. HPRG670, ThermoFisher). After initial culture and maturation, cells were exposed to serial dilutions of each API or vehicle/solvent control (1% DMSO) in William’s E medium supplemented with HepaRG™ Serum-free Induction Medium supplement (ThermoFisher, ref. HPRG750), with medium and treatment renewed every 2–3 days over a 14-day period. As a negative control (vehicle), cells were treated for the same duration with 1% DMSO. 1% Triton X-100 treated cells for the same incubation duration served as positive control. Each API was tested in biological triplicates, with at least three technical replicates per condition.

Cell viability, and the EC_50_, was measured using the CellTiter-Glo® 2.0 ATP assay (Ref. G9241, Promega, Madison, WI, USA) on Day 14 of treatment. After removing the medium, 50 µL of serum-free medium and 50 µL of reagent were added to each well, followed by shaking and incubation. Luminescence was measured using a plate reader, with results normalized to vehicle and positive controls.

Albumin and alanine aminotransferase (ALT) were measured using cell culture medium collected on days 3, 8, and 15. ALT (ref. ab234578, Abcam, Cambridge, United Kingdom) and albumin (ref. ab179887, Abcam) levels were quantified using commercial ELISA kits, with samples diluted as per manufacturer’s instructions. ALT concentration data were normalized to vehicle and positive controls. Results of albumin concentration were normalized to the vehicle controls and expressed as percentage.

### 2.5 Cytochrome P450 enzyme activity measurement

Cytochrome P450 (CYP) enzyme activity was assessed in HepaRG cells previously treated for 14 days with subtoxic concentrations of each API. After removal of the culture medium and washing with phosphate buffered saline (PBS), cells were incubated for 5 h at 37 °C with probe substrate cocktails prepared in serum-free medium. Two distinct cocktails were used to assess the activity of six major hepatic CYP isoforms:

- Cocktail A:
  o Phenacetin (104 μM; ref. 7,740, Merck; CYP1A2 probe)
  o Diclofenac (36 μM; ref. SML3086, Merck; CYP2C9 probe)
  o Testosterone (100 μM; ref. 86,500, Merck; CYP3A4 probe)
- Cocktail B:
  o Bufuralol HCl (36 μM; ref. UC168, Merck; CYP2D6 probe)
  o 7-Ethoxycoumarin (20 μM; ref. E1379, Merck; CYP2A6 probe)
  o(S)-(+)-Mephenytoin (100 μM; ref. UC175, Merck; CYP2C19 probe)

Following incubation, the culture medium from each well was collected and stored at −80 °C until analysis. Probe-specific cytochrome P450 metabolites were quantified using liquid chromatography–high-resolution mass spectrometry. Samples were extracted with ethyl acetate in the presence of isotopically labelled internal standards and analyzed on a reversed-phase C18 column using a 10-min water/acetonitrile gradient containing ammonium acetate and formic acid. Detection was performed on a Q-Exactive mass spectrometer (Thermo Scientific, Waltham MA, USA) operated in positive electrospray ionization mode.

Quantification was based on isotope-dilution using matrix-matched calibration curves with eight non-zero concentration levels and three quality-control levels. Calibration curves showed good linearity (*R* ^2^ ≥ 0.99). The method was developed for quantitative screening purposes and was not fully validated; however, limits of detection (LOD) and lower limits of quantification (LLOQ) were experimentally determined. LOD values ranged from approximately 1–16 ng/mL and LLOQ values from 3 to 52 ng/mL, depending on the metabolite. In addition, selectivity, linearity, accuracy & precision, and stability were successfully assessed prior to sample analysis.

For each CYP isoform, enzyme activity was determined by the formation of its specific metabolite and expressed as the ratio of metabolite concentration in API-treated wells relative to vehicle control wells. Only wells with ATP content ≥80% of untreated controls were included in the analysis to ensure data reflected viable cell populations.

### 2.6 Preparation and analysis of CBD dose formulations

API2 for animal dosing was prepared by dissolving 8.86 mg/mL in sesame oil (NF grade SE130, Spectrum chemical MFG CORP, Thermo Fisher Scientific). CBD concentration was determined using high-performance liquid chromatography (HPLC) with UV detection at 210 nm (Agilent Technologies, Inc. Santa Clara, California, USA). Calibration standards were prepared from a CBD stock solution in methanol:water (80:20, v/v), covering the validated range. Chromatographic separation was achieved on a Agilent C18 column using a mobile phase of methanol and deionized water (85:15, v/v) at 1 mL/min and 30 °C. Quantification was based on a linear calibration curve with acceptance criteria for selectivity, linearity (r ≥ 0.990), and accuracy. The LLOQ was 25 μg/mL, and the LOD was 5 μg/mL. The method was validated and determined to be fit for purpose based on selectivity, linearity, accuracy & precision, and stability assessments performed prior to sample analysis.

### 2.7 Animal selection and group assignment

Male Sprague Dawley rats were obtained from InVivos Pte. Ltd (Singapore). All animal housing and procedures were conducted at Verdeya Research Laboratories under institutional animal care and use protocol number A230001, in compliance with the National Advisory Committee of Laboratory Animal Research (NACLAR) Guideline (NACLAR, 2022) and Association for Assessment and Accreditation of Laboratory Animal Care (AAALAC) requirements.

Thirteen rats, selected randomly from the same age cohort by the animal breeder, were assigned to the dosing groups. Control plasma samples refer to the Time point at pre-dose or 0-h on Day 1. Control tissue samples refer to tissues extracted from non-dosed animals. To determine an appropriate sample size capable of providing statistically reliable results, a power calculation was performed using JMP software (JMP Statistical Discovery LLC, Singapore; version 16.1), assuming a two-sided hypothesis test. Based on existing AUC_last_ data (Sandmeier et al., 2025), it was determined that a group size of 13 rats per group is required to reliably detect a 40% difference between two independent groups with 80% power. This calculation was based on a two-sample t-test and a significance level (alpha) of 0.05.

Rats had *ad libitum* access to sterilized water. As feeding is known to impact CBD absorption (Feng et al., 2021a; Feng et al., 2021b) and to minimize inter-subject PK variability, rats were provided with standardized amounts of food (10 g/animal) on the day before Day 1 and Day 21, for 16–18-h. Following dosing (2–4 h) and for the rest of the study days, rats were provided *ad libitum* access with irradiated Teklad Global Rodent Diet 2914 C (Inotiv, West Lafayette, IN, USA).

### 2.8 Repeated oral dosing and bioanalysis

Rats (N = 13), 10 weeks old (body weight 365–405 g) at the time of initial dosing, were given a dose of CBD (26.5 mg/kg) in sesame oil by oral gavage once daily for 21 days. Dosing was performed using a flexible polypropylene feeding tube (18-gauge, Prime Bioscience, Singapore) fitted onto a syringe of appropriate size. The actual dose volume (mL/kg) was approximately 3 mL/kg and was based on the last measured body weight of each rat. Rats were weighed at least twice per week.

Blood samples were collected and processed into plasma samples according to a previous study (Sandmeier et al., 2025). Blood collections included a within 30 min pre-dose sample at intermittent days (days 2, 3, 7, 10, 14 and 17) during the 21-day period and in a series of time points following the first (Day 1 – 0-, 0.5-, 1-, 2-, 4-, 8-, 18-, 24-h) and last (Day 21, 0-, 0.5-, 1-, 2-, 4-, 8-, 18-, 24-, 48-, 72-h) oral dose for PK analysis. Liver, brain, lung, epididymal adipose tissue and mesenteric lymph nodes (MLN) were collected following the last blood draw and extracted with 2 volumes of 5 mM ammonium formate using the TissueLyser II (Qiagen, Hilden, Germany). Quantification of CBD, 6-OH-CBD, 7-OH-CBD, and 7-COOH-CBD in plasma and tissue extracts was performed using a liquid chromatography–tandem mass spectrometry method (Sandmeier et al., 2025). Briefly, following protein precipitation with acetonitrile, analytes were separated on a reverse-phase C18 column (Waters Pacific, Pte. Ltd., Singapore) and detected by positive electrospray ionization in multiple reaction monitoring mode on a Waters Xevo TQ Absolute mass spectrometer. Quantification was based on isotope-dilution using deuterated internal standards and linear calibration curves (1/x^2^ weighting). The assay was verified for linearity, accuracy, and precision over a concentration range of approximately 0.439–625 ng/mL.

### 2.9 Pharmacokinetics analysis

Pharmacokinetics profiles were evaluated by the non-sparse sampling approach of non-compartmental analysis using Phoenix WinNonlin (version 8.3.5; Certara, NJ, USA) according to previously published method (Sandmeier et al., 2025). Absolute bioavailability was calculated using area under the curve (AUC) data from intravenously administered synthetized CBD (Sandmeier et al., 2025).

### 2.10 Statistics

Statistical analyses were performed using JMP software package (JMP Statistical Discovery LLC, Singapore; version 16.1) with a significance level of p < 0.05 reported for all statistical tests. Geometric mean revertant per concentration, geometric standard deviation factor per concentration and revertant ratio of each tested dose vs. solvent were calculated for Ames assay. Geometric mean and geometric standard deviation factor of the percentage micronucleus positive cells (%MN) were calculated for the *in vitro* micronucleus assay. The %MN of the tests or positive controls versus concurrent vehicle-treated control were assessed using a one-sided Dunnett’s test. Trend test for both Ames and *in vitro* micronucleus assays was performed using the Kendall correlation (a positive correlation and *p* value ≤0.05 indicated positive test outcome).

Pairwise comparison of PK parameters was performed using two-sample Welch’s t-test. If the data showed an obvious departure from the normal distribution separately for the two groups being compared based on Shapiro-Wilk Test (p < 0.05), a non-parametric test was used (Wilcoxon rank-sum test).

For comparisons of analyte concentrations across multiple time points, the statistical approach commenced with an assessment of the assumption of normality. If normality was not satisfied initially, a log transformation was applied to the data, and the same sequence of checks was repeated. When normality was confirmed and sphericity held, a Repeated Measures ANOVA was conducted. If normality was satisfied but sphericity was violated, the *p-*values were adjusted using the Greenhouse–Geisser correction (GG). In cases where normality could not be achieved even after transformation, the Friedman test was employed. If the p-values indicated statistical significance, post hoc analyses were conducted using paired t-tests for data that met parametric assumptions, or Wilcoxon signed-rank tests for non-parametric data. To control false discovery rate, the Benjamini–Hochberg procedure was performed. Statistical significance was reported at three levels: p < 0.05, p < 0.01 and p < 0.001, as appropriate.

## 3 Results

### 3.1 *In vitro* genotoxicity

**FIGURE 1:**
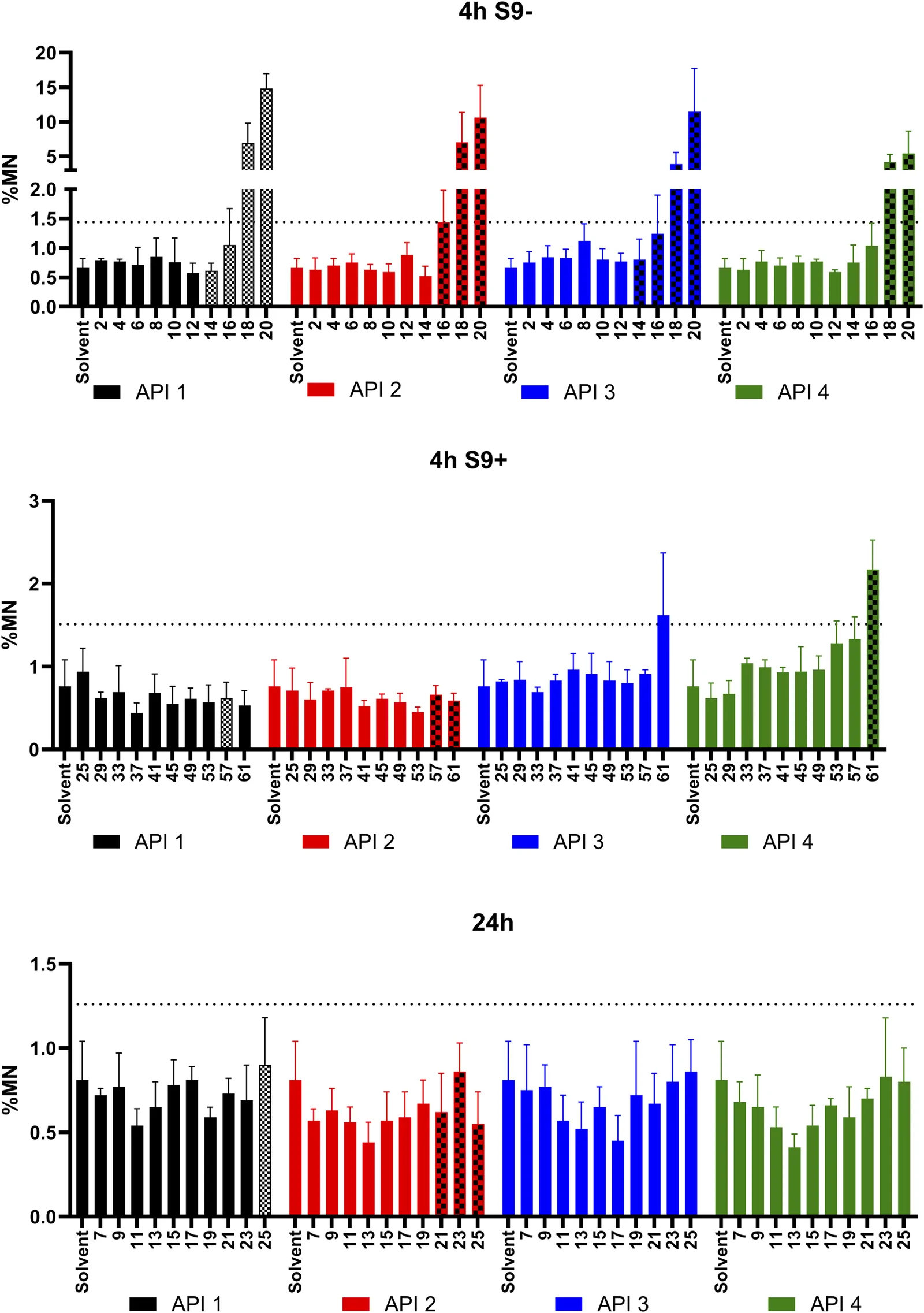
Frequency of micronucleated CHO cells (%MN) incubated with 4 APIs. The micronucleus assay was conducted using CHO-WBL cells exposed to three different experimental conditions: 4-h incubation without metabolic activation (4 h S9-), 4-h incubation with metabolic activation (4 h S9+), and a 24-h exposure. The values represent the mean and standard deviation of three replicates. Concentrations in µM are given in the x-axis. Bars with checkerboard patterns correspond to doses with a %RPD below 40 and therefore excluded from analysis. The dashed line corresponds to the upper range of the historical control range for the solvent.

Analysis of the four APIs (Figure 1) showed that none of the tested concentrations elicited a statistically significant or dose-dependent increase in micronucleus frequency (%MN). For API 3, a single concentration under the 4 h S9+ condition showed a %MN value above the historical control range; however, this increase was not statistically significant and was not associated with a dose-related trend. Therefore, it did not meet the predefined acceptance criteria for a positive response. Consequently, this isolated finding was considered not biologically relevant, and API 3, as well as API1, API2, and API4 were classified as non-genotoxic under the experimental conditions used.

In contrast, the positive controls demonstrated a statistically significant elevation in %MN compared to the solvent control group (Supplementary Table 1). Importantly, this increase remained within the historical range for positive controls, thereby confirming the sensitivity and responsiveness of the test system. These findings validate that the assay functioned as intended and that the negative results observed for the test concentrations are reliable within the experimental parameters.

Thus, none of the tested APIs are considered genotoxic in the conditions tested.

### 3.2 *In vitro* mutagenicity

The assessment of mutagenic potential using the Ames assay revealed that none of the doses tested for any of the four APIs produced an increase in revertant colony counts above the established strain-specific thresholds (Table 2). Furthermore, there was no observable trend indicating a dose-dependent rise in revertant colonies across the dose ranges evaluated. These findings indicate the absence of mutagenic activity for all four APIs under the experimental conditions employed.

**TABLE 2:** Ratio of revertant colony counts compared to the solvent-treated bacteria for five different *Salmonella Typhimurium* strains in the S9- and S9+ conditions.

​	TA98		TA100		TA102		TA1535		TA1537	
CBD (µM)	S9-	S9+	S9-	S9+	S9-	S9+	S9-	S9+	S9-	S9+
API 1										
1	1.0	1.0	0.6	1.2	1.0	0.7	1.0	1.3	1.3	1.1
25	0.9	0.9	0.6	1.2	0.9	0.9	0.8	1.3	1.5	0.8
50	0.8	1.0	0.6	1.0	0.8	0.9	0.8	0.9	0.5	0.9
75	1.2	1.1	0.5	0.9	0.8	0.9	1.0	1.5	1.1	1.1
100	1.0	1.0	0.8	1.1	0.8	1.0	1.0	1.4	0.5	1.3
124.29	0.9	0.9	0.7	1.1	0.7	0.9	0.7	1.0	0.6	0.9
API 2										
0.1	1.0	1.0	1.0	1.0	1.0	1.0	0.9	0.4	2.7	0.9
1	1.0	1.1	1.0	1.1	1.0	1.1	0.7	0.8	2.8	0.7
25	0.8	1.2	0.8	1.1	0.8	1.2	1.1	0.6	2.9	0.5
50	0.5	1.1	0.7	1.1	0.5	1.1	1.2	1.0	2.0	0.7
75	0.6	1.1	0.7	1.0	0.6	1.1	0.8	0.9	1.0	0.7
124.29	0.6	1.0	0.7	1.0	0.6	1.0	0.7	0.8	1.9	0.7
API 3										
1	0.7	1.0	1.1	1.0	0.8	0.9	0.7	1.2	1.6	2.0
25	0.8	1.1	1.1	1.0	0.8	1.0	0.7	0.7	2.3	1.8
50	1.1	1.3	1.0	0.8	1.0	1.0	1.0	0.5	1.9	1.8
75	0.6	1.1	1.1	0.9	0.8	0.9	0.8	0.9	0.9	2.1
100	0.6	1.2	0.9	0.9	1.0	1.0	1.1	1.0	0.9	1.9
124.29	0.8	1.1	0.9	0.7	0.8	0.9	0.7	0.9	1.2	1.5
API 4										
1	1.2	1.1	0.9	1.0	1.1	1.1	0.9	1.3	1.1	1.0
25	1.1	1.0	0.6	0.9	0.9	1.0	0.8	0.9	1.1	1.1
50	1.1	1.2	0.8	0.7	0.8	1.1	0.7	1.5	0.7	1.3
75	1.0	1.1	1.0	0.7	0.7	0.9	0.6	1.1	0.5	1.2
100	0.8	1.0	1.0	0.6	0.6	1.0	0.8	1.5	0.4	1.2
124.29	1.0	1.2	0.9	0.6	0.7	1.0	0.8	1.3	0.9	1.4

The Ames assay was conducted utilizing five distinct strains of *Salmonella typhimurium*: TA98, TA100, TA102, TA1535, and TA1537. Each strain was tested under both S9- (without metabolic activation) and S9+ (with metabolic activation) conditions to thoroughly assess mutagenic potential. Each ratio is based on the colony count of 3 replicates per dose.

In contrast, the positive control samples demonstrated the expected sensitivity of the bacterial strains to mutagenic agents, as evidenced by an increase in revertant colonies above the respective threshold values for each strain (The positive control must induce a 2-fold (TA98, TA100, and TA102) or 3-fold (TA1535 and TA1537) increase in revertant colony count when compared to the solvent control colony count; Supplementary Table 2). This outcome confirmed the responsiveness and validity of the assay system.

All experimental runs met the acceptance criteria for both bacterial viability and sterility (data not shown), in accordance with the guidelines established by the OECD. Based on these results, it was concluded that all four APIs tested are non-mutagenic in the Ames assay conditions applied.

### 3.3 *In vitro* liver cytotoxicity

**FIGURE 2:**
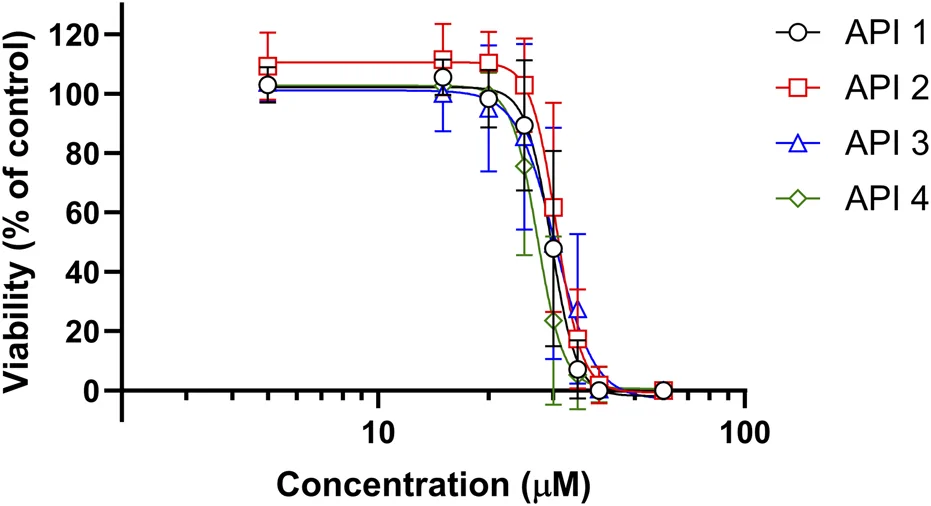
HepaRG viability measurement following a repeated 14-day exposure to a dose range of the 4 APIs. Each API was tested over eight concentrations ranging from 5 to 60 µM. Each dot is the mean ± standard error of nine replicates. 100% corresponds to the viability of the solvent control. The viability was normalized to cells treated with 1% Triton X-100 for 24 h (0%) and to cells treated with the solvent (100%).

**FIGURE 3:**
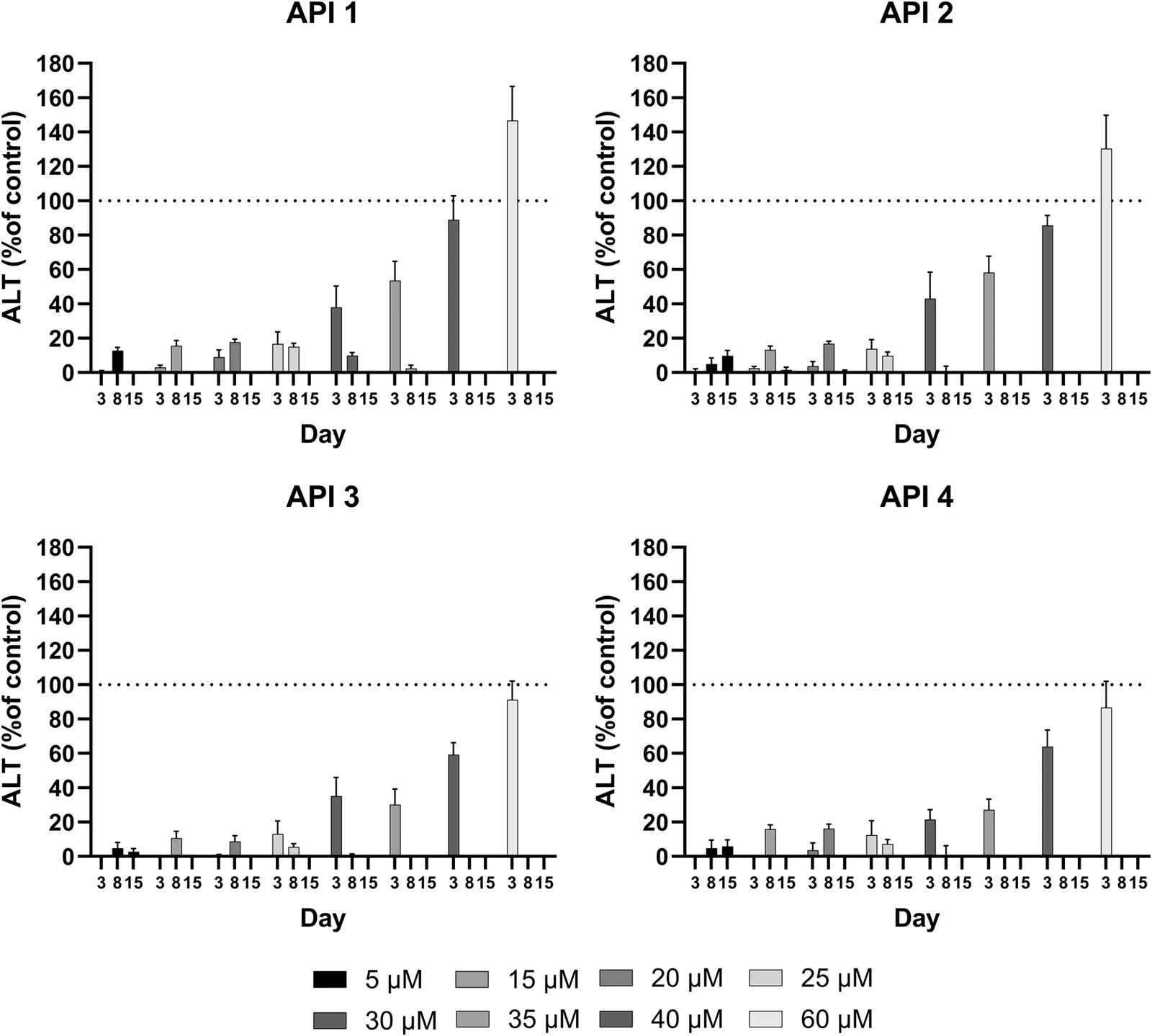
ALT concentration following a repeated 14-day exposure to a dose range of the 4 APIs. The level of ALT present in the cell culture medium was measured on days 3, 8, and 15 following treatment. Each bar is the mean ± standard error of nine replicates. 100% corresponds to the ALT released by cells treated with 1% Triton X-100 for 24 h, and 0% to the ALT released by cells treated with the solvent. The ALT concentration was normalized to cells treated with 1% Triton X-100 for 24 h (100%) and to cells treated with the solvent (0% ALT).

**FIGURE 4:**
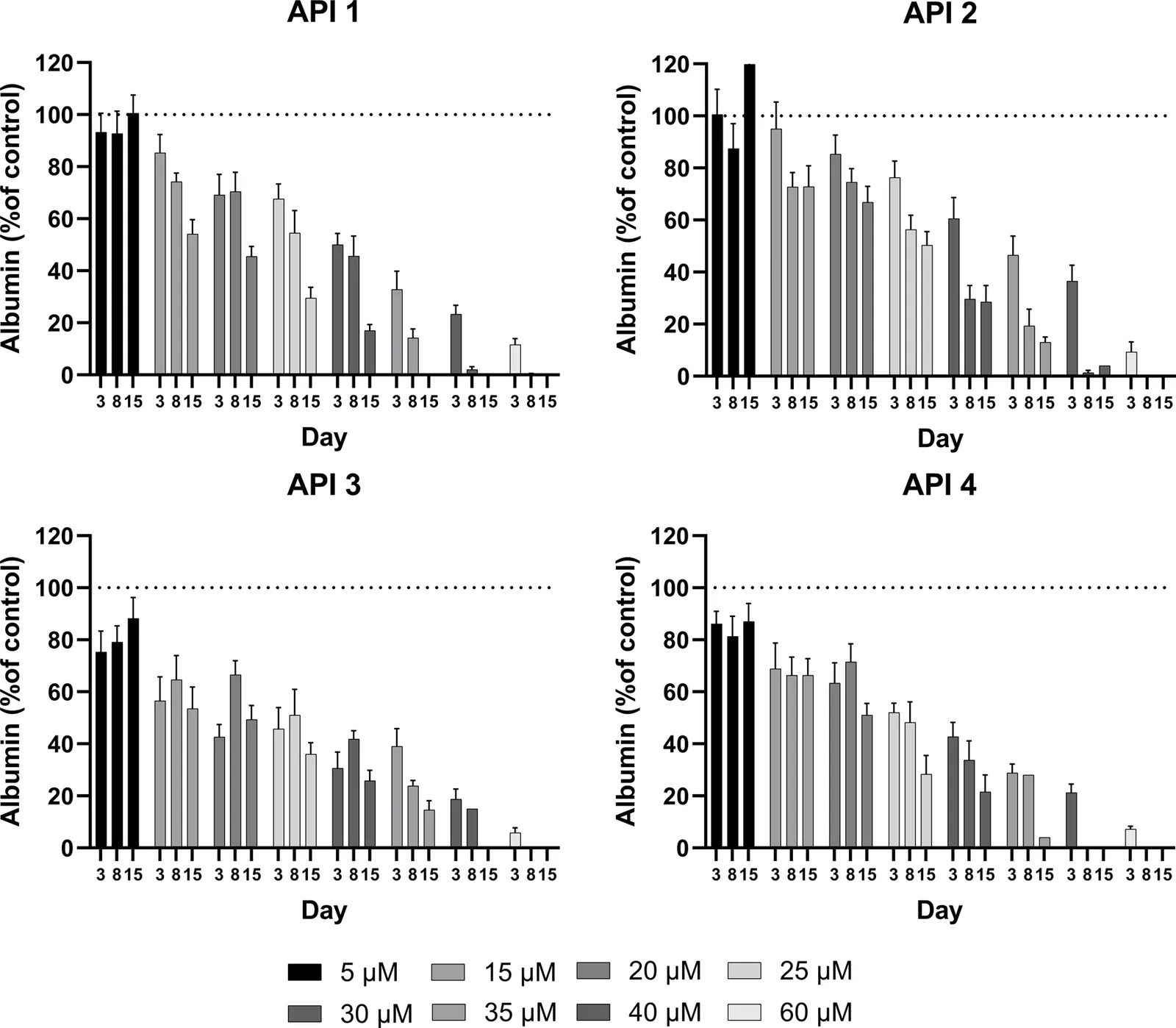
Secreted albumin following a repeated 14-day exposure to a dose range of the 4 APIs. The cell culture medium was assessed for albumin concentration on days 3, 8, and 15. Each bar is the mean ± standard error of nine replicates. 100% corresponds to the albumin secreted by cells treated with the solvent. The albumin concentration was normalized to cells treated with the solvent (100% albumin).

In the HepaRG liver model, all four CBD samples demonstrated virtually identical hepatotoxicity profiles (Figure 2). The cytotoxic potency (EC_50_) measured using the intracellular ATP level was ∼27–30 µM for each API, with very similar steep dose–response curves. High concentrations of any of the four CBD samples (above ∼30 µM) produced the same pattern of acute cell injury: an early spike in ALT release ∼48 h after exposure (indicating cell membrane damage and hepatocellular injury), followed by a return to baseline at later time points as severely damaged cells were lost (Figure 3). All four APIs at cytotoxic doses also caused a >50% reduction in albumin secretion by the HepaRG cells (Figure 4). Notably, even at sub-cytotoxic concentrations (e.g., 15 μM), each API caused a similar 20%–40% decrease in albumin production, suggesting a consistent functional impact of CBD on hepatocytes independent of overt cell death.

### 3.4 *In vitro* CYP activity

**FIGURE 5:**
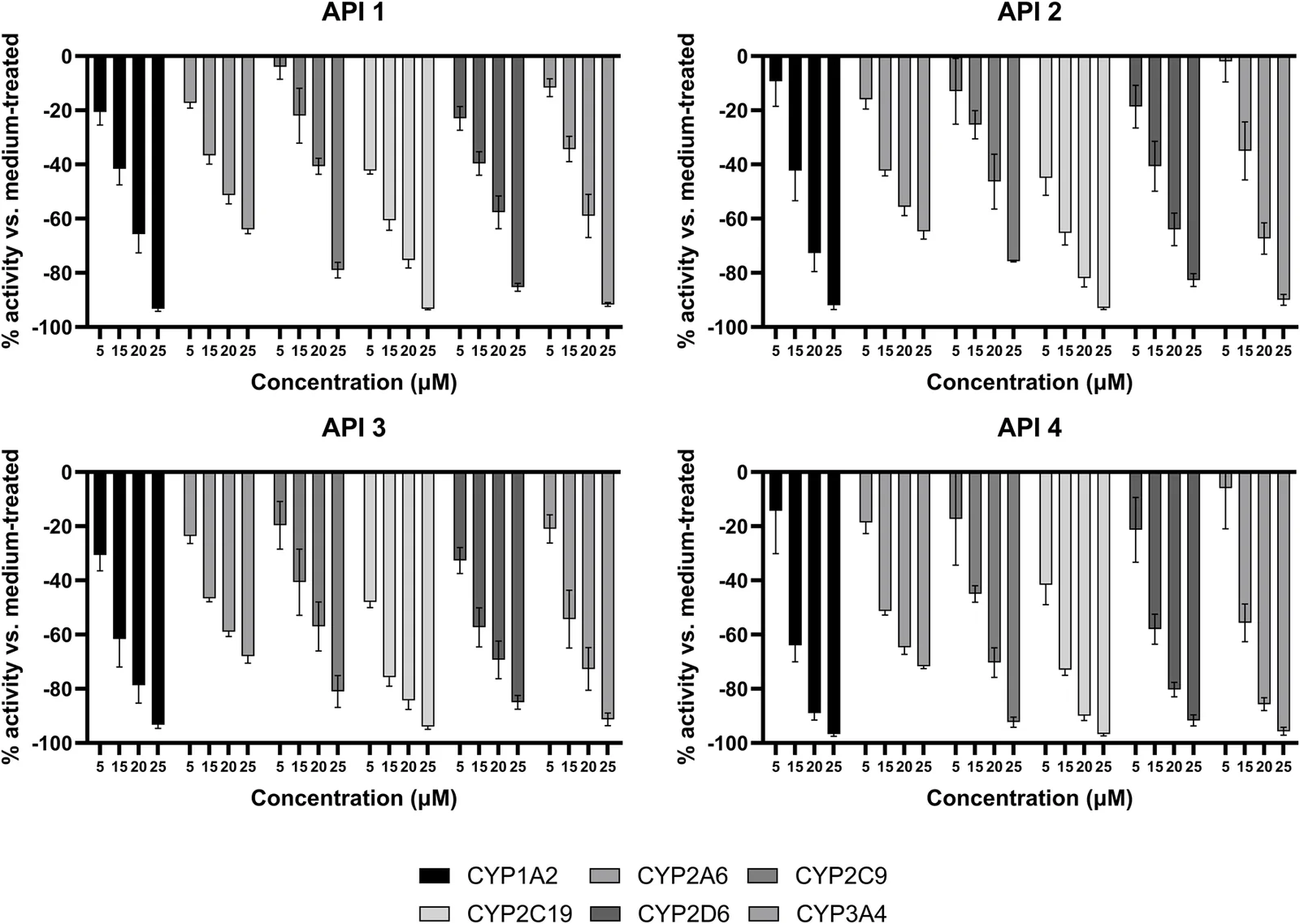
Change in CYP activity in HepaRG cells exposed repeatedly for 14 days to a dose range of the 4 APIs. CYP1A2, 2A6, 2C9, 2C19, 2D6, and 3A4 activities were measured using CYP specific probes. Each bar is the mean ± standard error of nine replicates. CYP activity changes are reported relative to solvent-treated cells (set as 0% activity).

The four APIs assessed demonstrated a dose-dependent reduction in the activities of CYP1A2, CYP2A6, CYP2C9, CYP2C19, CYP2D6, and CYP3A4 (Figure 5). Enzyme activity decreased proportionally with increasing concentrations of the APIs. At the lowest concentration tested (5 µM), reductions of up to 30% were observed for CYP2C19 and CYP2D6. At the highest concentration (25 µM), all six enzymes exhibited a decrease in activity ranging from 65% to 95% compared to untreated controls. Among the enzymes, CYP2A6 displayed the least reduction across all APIs, whereas the remaining five enzymes showed comparable declines at each dose level. No significant differences in enzyme activity were noted when comparing the effects of individual APIs.

### 3.5 Accuracy of dose formulations

The comparative *in vitro* assessment did not identify meaningful API-dependent differences among the four CBD materials in mutagenicity, genotoxicity, HepaRG cytotoxicity, hepatic functional endpoints, or CYP activity. Therefore, API2, a GMP-grade synthetized CBD API with high purity, was selected as a representative material for the *in vivo* PK study. The *in vivo* PK arm was designed as an exposure-contextualization study to support the *in vitro* findings through exposure and accumulation characterization while in alignment with the 3R (replacement, reduction and refinement) principles in animal research. The measured CBD concentrations in dosing formulation for API2 (synthetized CBD) are based on analytical quantification of CBD in the prepared formulations and are summarized in Supplementary Table 3. The measured CBD concentrations closely match the theoretical value, demonstrating both precision and consistency in the formulation and analysis processes. The delivered CBD doses normalized to body weights are in Supplementary Table 4. The rats were accurately dosed according to their body weights with achieved delivered CBD doses of 26.469 mg/kg and 26.514 mg/kg on dosing Day 1 and Day 21, respectively.

### 3.6 In-life observations and CBD uptake following oral administrations

**FIGURE 6:**
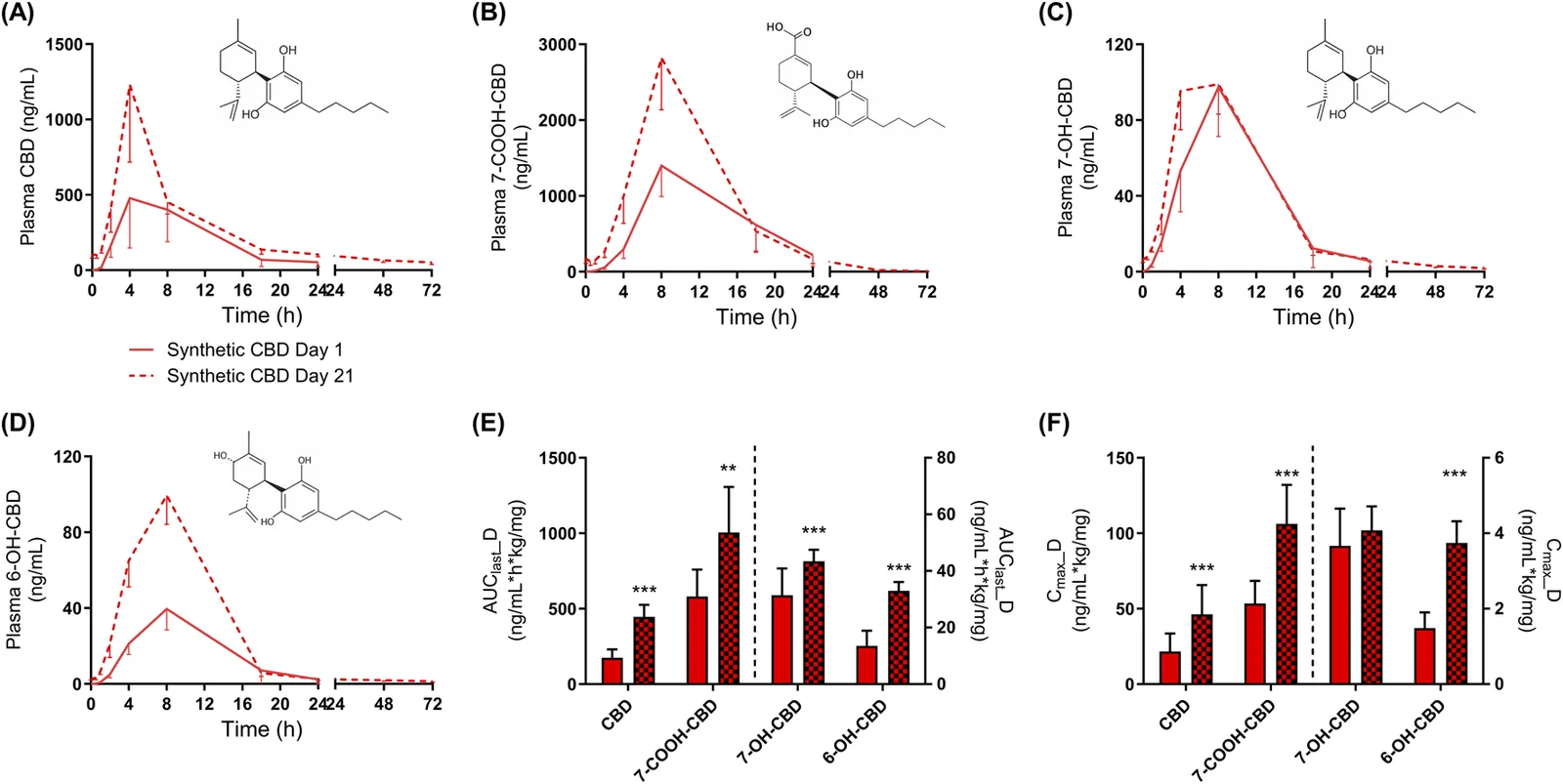
Pharmacokinetic profiles of CBD, 7-COOH-CBD, 7-OH-CBD and 6-OH-CBD upon repeated dosing of API 2. Data shown are plasma concentration vs. time plots with mean and standard deviation of **(A)** CBD, **(B)** *7*-COOH-CBD, **(C)** 7-OH-CBD and **(D)** 6-OH-CBD collected during Day 1 and Day 21 following oral dosing. The chemical structures corresponding to CBD and its metabolites are shown in the top right corner of **(A)** to **(D)**. The PK parameters of CBD and its metabolites are shown for **(E)** dose-normalized area under the curve (AUC_last__D) and **(F)** maximum plasma concentration (C_max__D). N = 13 male rats. Bars with filled and checkerbox pattern refer to data from day 1 and day 21, respectively. ** and *** refer to statistical significance at p < 0.01 and p < 0.001, respectively, at Day 21 versus Day 1. LLOQ is same for CBD, 7-COOH-CBD, 6-OH-CBD and 7-OH-CBD at 0.439 ng/mL.

There were transient weight losses (∼2–3%) noted on day 3 and day 24 relative to the prior measured averages, likely attributed by multiple blood draws. Otherwise, the rats gained weight progressively (∼12%) throughout the study with no other notable clinical observations (Supplementary Table 5). Quantification of the plasma levels of CBD confirmed the uptake into systemic circulation following repeated oral administration (oral gavage) of CBD formulation (Figure 6). The average concentrations of CBD and its metabolites in the Day 1 pre-dose samples were below the LLOQ.

### 3.7 Pharmacokinetics profiles of CBD and metabolites

CBD was absorbed from the gastrointestinal tract and detectable concentrations were still observable in plasma up to 24-h post-dose on Day 1 and 72-h post-dose on Day 21. Maximum CBD concentrations could be reached at average 5.5 h (range 4- to 8-h) and 4.0 h post-administration on Day 1 and Day 21, respectively (Figure 6; Table 3). There were (statistically significant) increases of dose-normalized AUC_inf_, AUC_last_, and C_max_ by 3.1-, 2.9, and 2.5-fold at Day 21 following repeated administration as compared to Day 1 (Supplementary Table 6). The half-life of CBD on Day 1 was 5.9 h (range 4.3- to 12.1-h), while the terminal elimination half-life was 41.9 h (range 24.3- to 60.4-h) on Day 21. These correspond to apparent clearance of 1836 mL/h/kg and elimination rate constant of 0.017 h–1 on Day 21.

**TABLE 3:** Average plasma CBD pharmacokinetic parameters following repeated oral dosing of API2.

​	​	​	Day 1		Day 21	
PK parameter	Units	N	Mean ± SD	%CV	Mean ± SD	%CV
AUC_inf_	h*ng/mL	8	5,555 ± 1,219	22	15,078 ± 3,299	22
AUC_inf_/Dose	h*kg*ng/mL/mg	8	209 ± 46	22	567 ± 124	22
AUC extrapolation	%	13	9.5 ± 9.7	N/A	20.8 ± 5.5	N/A
AUC_last_	h*ng/mL	13	4,649 ± 1,493	32	11,844 ± 2,137	18
AUC_last_/Dose	h*kg*ng/mL/mg	13	175 ± 56	32	446 ± 80	18
C_max_	ng/mL	13	577 ± 316	55	1,231 ± 515	42
C_max_/Dose	kg*ng/mL/mg	13	22 ± 12	55	46 ± 19	42
T_max_	h	13	5.5 ± 2.0	37	4.0 ± 0.0	0
T_last_	h	13	24.0 ± 0.0	0	72.0 ± 0.0	0
T_1/2_	h	8	5.9 ± 2.6	44	41.9 ± 9.7	23
CL/F	mL/h/kg	8	4,975 ± 1,023	21	1836 ± 371	20
K_e_	1/h	8	0.13 ± 0.031	24	0.017 ± 0.004	26
Vz/F	mL/kg	8	40,820 ± 13,772	34	108,657 ± 24,081	22
Bioavailability	%	13	25.6	N/A	N/A	N/A

Data shown are mean and standard deviation (SD) of the individual PK, profiles with coefficient of variation %CV) indicated. N, number of independent data; AUC, area under the concentration versus time curve reported as AUC_last_ - 0, to last measurable time point, and AUC_inf_, 0 to infinity time point; CL/F, apparent clearance or volume of blood that has CBD, removed per unit of time; C_max_, maximum observed concentration; K_e_, terminal phase elimination rate constant or fraction of CBD removed per unit of time; T_max_, time after dosing at which the maximum observed concentration was observed; Vz/F, apparent volume of distribution during terminal phase or volume into which the drug distributes in the body: N/A, not applicable.

**FIGURE 7:**
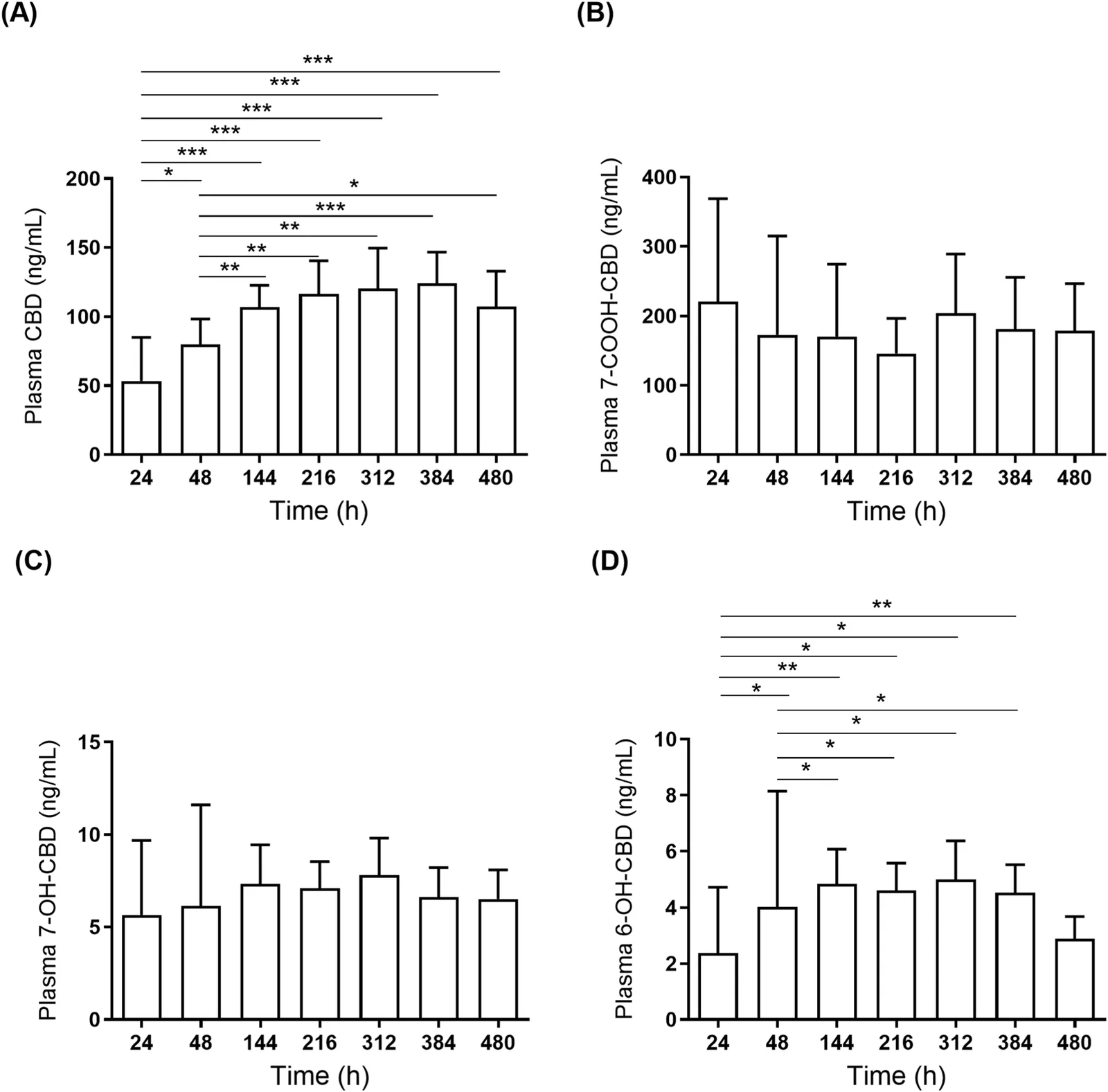
Pre-dose plasma concentrations of CBD, 7-COOH-CBD, 7-OH-CBD and 6-OH-CBD during repeated oral administration of API 2. Data shown are mean and standard deviation of plasma concentrations of **(A)** CBD, **(B)** 7-COOH-CBD, **(C)** 7-OH-CBD and **(D)** 6-OH-CBD collected pre-dose during the 21-day dosing period. The 24-h, 48-h, 144-h, 216-h, 312-h, 384-h and 480-h time points refer to 1 day, 2 days, 6 days, 9 days, 13 days, 16 days and 20 days, respectively, following the initial dosing. Differences between time points are indicated with *p < 0.05, **p < 0.01 or ***p < 0.001. N = 13 male rats.

The CBD trough concentrations were measured at 24-h, 48-h, 144-h, 216-h, 312-h, 384-h and 480-h time points following the first oral dosing. A statistical approach was used to compare the plasma CBD trough concentrations between one time point to the later time points. Statistically significant differences in CBD trough concentrations were noted at 24-h and 48-h compared to the later time points, reflecting gradual systemic accumulation of CBD after the 24-h and 48-h time points. On the other hand, there were no statistically significant differences in plasma CBD concentration between 144-h and the subsequent time points. Therefore, the estimated average CBD trough concentration (107 ng/mL) in the plasma was deemed to no longer increase statistically significantly after 6 days of repeated dosing (Figure 7). The maximum plasma concentrations (C_max_) achieved on Day 1 and Day 21 were approximately 5-fold and 12-fold higher than the trough concentration (Table 3).

The CBD was converted to its metabolites, 7-COOH-CBD, 7-OH-CBD and 6-OH-CBD, with detectable plasma concentrations of the metabolites mostly observed within 24-h post-dose on Day 1 and 72-h post-dose on Day 21 (Figure 6; Supplementary Table 6). Maximum 7-COOH-CBD and 6-OH-CBD concentrations were observed mostly at 8-h post-dose on days 1 and 21, while maximum 7-OH-CBD concentrations were observed at 8-h post-dose on Day 1 and 4–8-h post-dose on Day 21. The most abundant metabolite, 7-COOH-CBD, displayed about 2- to 3-fold higher AUC and C_max_ than CBD in the plasma on Day 1 and upon repeated dosing (Supplementary Table 6). The 7-OH-CBD metabolite was present at only 10%–20% of that of CBD levels on Day 1 and upon repeated dosing, while 6-OH-CBD was 8%–10% of CBD levels on Day 1 and upon repeated dosing (Supplementary Table 6). Repeated oral dosing also resulted in statistically significant (approximately 2-3-fold) increase in plasma 7-COOH-CBD and 6-OH-CBD exposure (dose-normalized AUC and C_max_) compared to single dosing (Supplementary Table 6). The exposure level in terms of AUC for 7-OH-CBD was 1.5-fold higher in repeated compared to single dosing. As with CBD, the trough concentrations for 6-OH-CBD showed statistically significant differences at 24-h and 48-h compared to the later time points, reflecting gradual systemic accumulation upon repeated dosing (Figure 7). Since there were no statistically significant differences in plasma concentration from 144-h, the 6-OH-CBD trough concentration was estimated at 4.8 ng/mL. The trough concentrations of 7-COOH-CBD and 7-OH-CBD did not show obvious change over the study period examined.

### 3.8 Tissue accumulation of CBD and metabolites

**FIGURE 8:**
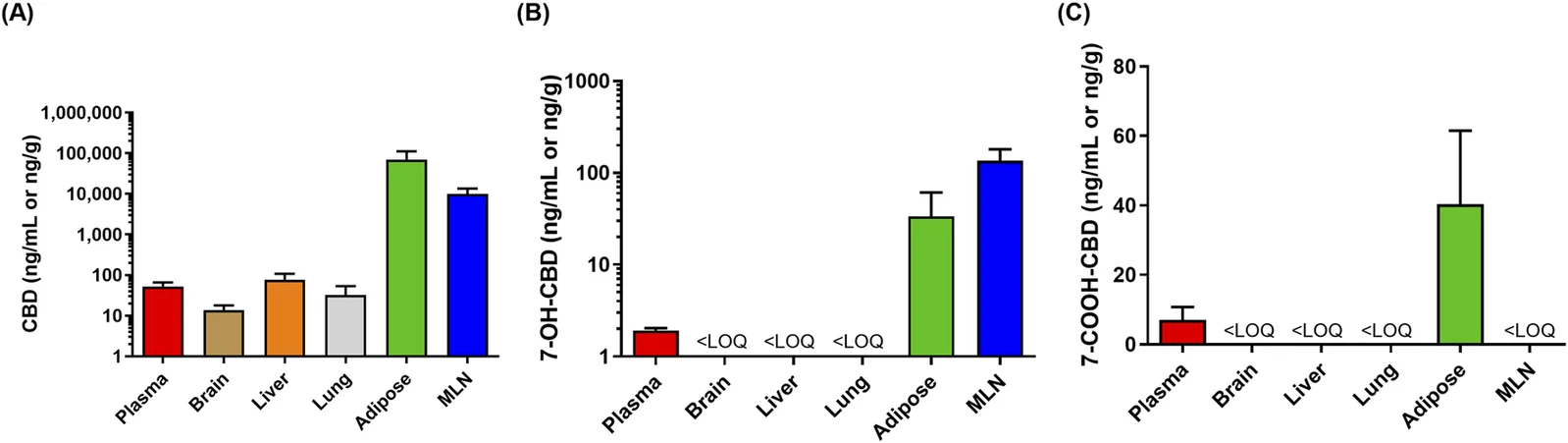
Tissue concentrations of CBD and its metabolites following oral dosing of API 2*.* Data are presented as mean and standard deviation. The plasma and tissue concentrations of **(A)** CBD, **(B)** 7-OH-CBD and **(C)** 7-COOH-CBD are shown. N = 13 male rats. MLN, mesenteric lymph nodes; LOQ, limit of quantification.

CBD and its metabolites were not detected in the tissues of non-dosed animals. Following repeated dosing, CBD was most abundantly detected in adipose tissue and MLN as compared to the brain, liver and lung (Figure 8; Supplementary Table 7). The tissue concentrations of CBD were 70,124 ng/g, 10,018 ng/g and 77 ng/g in the adipose tissue, MLN and liver, respectively. The tissue to plasma concentration ratios of CBD were 1,470, 201 and 1.5, in the adipose tissue, MLN and liver, respectively, indicating preferential accumulation of CBD in these tissues. There was no obvious CBD accumulation in the brain or lung tissues (tissue to plasma concentration ratios less than 1). Of the 3 CBD metabolites, only 7-OH-CBD (42 ng/g) and 7-COOH-CBD (51 ng/g) were measurable in small quantities in the adipose tissue, and 7-OH-CBD (136 ng/g) was also detected in the MLN. The adipose tissue to plasma concentration ratios of 7-OH-CBD (20.9-fold) and 7-COOH-CBD (10-fold), as well as MLN tissue to plasma concentration ratio of 7-OH-CBD (73.4-fold) suggest accumulations in the adipose tissue and MLN.

## 4 Discussion

This study provides a head-to-head comparison of synthetized and plant-derived CBD, assessing whether CBD source and purity influence *in vitro* genotoxicity and hepatotoxicity, and characterizing accumulation and tissue distribution after repeated oral dosing in rats.

Our finding of no genotoxicity or mutagenicity for CBD agrees with a broad consensus of prior studies. Multiple independent, GLP-compliant investigations–using high-purity CBD from synthetized and plant sources–have uniformly reported negative results in standard genotoxicity assays. For example, Henderson et al. (Henderson et al., 2023b) tested a ≥99% pure CBD isolate in the OECD 471 Ames bacterial reverse-mutation assay (five *Salmonella* strains and *E. coli* WP2) at doses up to 5,000 µg/plate and observed no increase in revertant colonies (no mutagenic activity) in any strain, with or without metabolic activation. The same study also evaluated CBD in an OECD 487 *in vitro* micronucleus test using human TK6 lymphoblastoid cells (up to 30–35 µM) and found no induction of micronuclei above vehicle-control levels. Tallon et al. (Tallon et al., 2025) similarly reported no genotoxic effects of a high-purity plant-derived CBD: in their study, a 97% CBD isolate (in MCT oil) showed no mutagenicity in Ames tests and no clastogenicity in an *in vitro* micronucleus assay, with no statistically significant increase in micronucleated cells at any dose. These rigorous studies, as well as our study–all following OECD guidelines–provide clear evidence that CBD is non-mutagenic in bacteria and non-genotoxic in human and Chinese hamster cells, reinforcing that CBD lacks intrinsic genotoxic potential in standard test systems. Early safety assessments of CBD-rich hemp extracts are consistent with these findings as well. For instance, Marx et al. (Marx et al., 2018) evaluated a supercritical CO_2_ extract of hemp (primary constituent CBD) and found no genotoxicity in the Ames assay or in mammalian chromosomal-aberration tests. Likewise, Dziwenka and colleagues (Dziwenka et al., 2020) reported that a broad-spectrum hemp oil (containing high levels of CBD) was negative for mutagenicity and clastogenicity, with no DNA damage observed *in vitro* and no micronucleus induction in rodents. More recently, Clewell et al. (Clewell et al., 2023) tested a CBD-rich hemp oil (∼20–30% CBD) and also found no evidence of genotoxicity: the oil did not induce mutations in Ames bacterial assays and did not increase micronuclei in human peripheral blood lymphocytes.

The overall pattern across these diverse preparations–from purified CBD isolates to natural extracts–is that CBD consistently yields negative results in genotoxicity test batteries, irrespective of source or minor impurities. This convergence of evidence suggests that CBD itself does not damage genetic material or cause mutations in standard *in vitro* systems. Taken together, our results align perfectly with this extensive body of evidence. Across disparate laboratories and test systems, CBD has not shown any genotoxic or mutagenic activity under experimental conditions compliant with international guidelines.

The *in vitro* hepatotoxicity assessment using HepaRG cells revealed a consistent and steep dose-dependent cytotoxic profile across all four APIs, regardless of their source (synthetized vs. plant-derived) or manufacturing grade (GMP vs. food-grade). After 14 days of repeated exposure, all CBD variants demonstrated similar EC_50_ values (∼27–31 µM), with no discernible differences in potency or toxicity patterns. This uniformity suggests that the hepatotoxic potential of CBD is intrinsic to the molecule itself and not significantly influenced by its origin or purity. The steepness of the dose-response curves indicates a narrow cytotoxicity window, with a rapid transition from minimal to complete cell death over a small concentration range. This threshold-like behavior was further supported by the temporal dynamics of cellular injury markers: ALT levels spiked within 48 h of exposure to concentrations at or above the EC_50_, then declined on subsequent days (days 8 and 15), consistent with early-onset cytotoxicity. Such kinetics are characteristic of apoptotic cell death rather than cumulative necrosis. This is in line with previous studies demonstrating CBD-induced apoptosis via cannabinoid receptor activation (Jeong et al., 2019; Greenwood et al., 2024), and the expression of cannabinoid receptors in HepaRG cells (De Nunzio et al., 2023) further supports this mechanism.

Pre-clinical animal research has also provided valuable data for assessing the safety of CBD with varying purities. A majority of the repeated dose oral safety toxicology studies range from 14 to 90 days in duration with additional post-exposure recovery period. Animals were dosed up to a high dose of approximately 150 mg/kg/day (Henderson et al., 2023a; Schwotzer et al., 2025; Xia et al., 2025), though one study dosed up to 460 mg/kg/day (Tallon and Child, 2023), all using high purity (>99%) CBD isolates. Despite being independent tests, the results are remarkably similar. Most endpoints were unaffected by the CBD treatment up to the high dose. In-life findings (body weight, food consumption, overall health status of the test system) were similar and demonstrated overall acceptable tolerance to the treatments. Organ weight changes were observed and were mainly stress-dependent effects such as higher weight of the adrenals and thyroid, with reduced weight of the spleen as compared to the control groups. There was consistently higher liver weight among the studies, also associated hepatocyte hypertrophy. These findings recovered following the post-exposure recovery period in all cases and were consistent with xenobiotic-induced microsome enzyme induction (Hall et al., 2012). Hence, these macro- and microscopic findings were considered as adaptive changes of no toxicological relevance by the pathologist. As comparison, when less purified hemp extracts were evaluated, slight differences in the overall no-observed-adverse-effect-levels (NOAELs) were reported (Marx et al., 2018; Dziwenka et al., 2020).

Importantly, the study also revealed that sub-lethal concentrations of CBD in vitro—again, independent of source or purity—impaired key hepatic functions. Albumin synthesis and secretion were significantly reduced at concentrations well below the cytotoxic threshold. At 15–20 μM, where ATP levels remained near baseline, albumin secretion dropped by 20%–60% compared to solvent controls. This functional impairment, in the absence of overt cytotoxicity, suggests that CBD may interfere with protein synthesis or secretion pathways. Supporting this hypothesis, Kosgodage et al. (Kosgodage et al., 2018) reported that CBD inhibits exosome and microvesicle release in cancer cells, a mechanism that could similarly affect albumin export in hepatocytes.

In parallel, CBD exposure led to a marked, concentration-dependent suppression of cytochrome P450 (CYP) enzyme activities. All six major CYP isoforms tested (CYP1A2, 2A6, 2C9, 2C19, 2D6, and 3A4) exhibited dose-dependent inhibition, with no observable differences between the four CBD APIs. Even at 5 µM—well below cytotoxic levels—enzyme activities were reduced by 5%–30%, and at 25 μM, inhibition reached 60%–95%. These findings are consistent with prior reports of CBD as a potent, reversible inhibitor of multiple CYP isoforms (Stout and Cimino, 2014; Nasrin et al., 2021). The reversibility of this inhibition, as demonstrated by the recovery of CYP activities upon CBD removal, suggests a non-permanent, likely competitive mechanism. While our study observed dose-dependent inhibition of major CYP isoforms at micromolar concentrations, other reports suggest that lower, nanomolar concentrations or repeated CBD dosing in humans can lead to enzyme induction, at least for specific CYPs such as CYP3A4 (Morrison et al., 2018; Balhara et al., 2025). These findings could imply that CBD’s modulation of hepatic metabolism may follow a biphasic pattern, with inhibitory effects predominating at acute high concentrations and possible induction at lower, and repeated dosing, but for substantiation, additional translatable data will be needed.

Mechanistically, the data indicates that the CBD APIs, regardless of their manufacturing process or botanical origin, possess a comparable cytotoxic potency in human liver cells *in vitro* as well as the potential to decrease albumin secretion and inhibit the activity of major CYP enzymes. The consistency of these effects across all four APIs underscores that the effects observed are a fundamental property of CBD itself, rather than a consequence of impurities or origin. Although the *in vitro* hepatotoxicity assessments demonstrated potential effects of CBD, consistent with findings from other studies (Li et al., 2023; Chen et al., 2024), such effects were only observed at very high concentrations within a narrow dose-response window, and their toxicological significance under physiological conditions remains to be fully elucidated. In human subjects repeatedly administered exceptionally high doses of CBD (1,500 mg/day), the plasma concentrations achieved under both fasted and fed conditions were 1.07 µM and 5.18 µM, respectively (Taylor et al., 2018). The reported plasma concentration of 5.18 µM in humans following high-dose oral CBD administration is substantially lower than those required in our study to induce hepatotoxic effects in cultured liver cells: the *in vitro* EC_50_ values (∼27–31 µM) are equivalent to 7,819–8,977 mg/day CBD in human, the *in vitro* sub-cytotoxic concentration (e.g., 15 μM) with about 50% decrease of albumin is equivalent to 4,344 mg/day CBD in human. Possible explanations for the observed *in vitro* “toxicity” include: (i) accumulation of metabolites such as 7-COOH-CBD in the static assay system, (ii) the absence of organotypic architecture and lack of metabolic support from communicating cell types present *in vivo*, and/or (iii) the use of exaggerated concentrations of CBD and its metabolites which is inherent to the typical design of *in vitro* assays, i.e., the need to increase the dosing until a substantial effect is achieved. In this context, it is also crucial to consider the potential for CBD accumulation during repeated administration, which could result in elevated plasma concentration over time. Additional risk factors include high CBD usage (≥1,000 mg/day or ≥20 mg/kg/day) and concomitant antiepileptic drug use (Lo et al., 2023), pediatric population, individuals with pre-existing liver impairment, elderly patients and polypharmacy as well as other factors that could impact the pharmacokinetics of CBD.

In this study, we used the same CBD source and bioanalytical method as the reported intravenous data (Sandmeier et al., 2025). These methodological approaches minimize variability due to CBD products or assay differences, thereby strengthening the validity and reliability of the bioavailability calculation. Under fed state and current study set-up, the CBD bioavailability is 25.6%. FDA/EMA-approved CBD formulation (100 mg/mL oral solution in a sesame-oil-based vehicle) had an estimated bioavailability of 6% under fasting state in humans (Millar et al., 2018; Perucca and Bialer, 2020). Co-administration with high-fat meals resulted in improved bioavailability compared with fasting state. This is demonstrated by the higher peak plasma concentration (C_max_) and the overall exposure (AUC) when CBD was taken with food (Taylor et al., 2018; Bergeria et al., 2022; Jang et al., 2024; Saals et al., 2025). This effect is likely due to lipid digestion, action of bile and enzymes for micelle formation which can improve dissolution of CBD and lymphatic transport (Charman et al., 1997; Birnbaum et al., 2019). Furthermore, experimental PK study in rats demonstrated higher CBD systemic bioavailability in lipid-based excipients compared to formulations without lipids with increased absorption of CBD in the presence of lipids by intestinal lymphatic transport (Zgair et al., 2016). In male rats under fed conditions (Supplementary Figure 1), the dose-normalized C_max_ and AUC_last_ values observed after oral administration of synthetized CBD in sesame oil were comparable to those obtained with hemp-derived CBD isolate formulated in olive oil (Xia et al., 2025). These results demonstrate that highly purified CBDs exhibited similar *in vivo* pharmacokinetic profiles following oral administration, regardless of whether it is synthetized or derived from hemp. The potential influence of other cannabinoids, e.g., in distillates, on PK of CBD needs to be further explored.

Consistent with the impact of food and lipid-based formulations to improve bioavailability of CBD, there was approximately 2-fold higher AUC_inf_ value in our study conducted under fed state compared to that of Feng et al. (Feng et al., 2021a; Feng et al., 2021b) where CBD in sesame oil formulation was administered to fasted rats. Our results are also consistent with higher bioavailability of sesame oil-based formulations compared to those containing medium-chain fatty acid (capric acid, C10:0) (Kok et al., 2022), potentially due to more efficient incorporation into chylomicrons for lymphatic uptake (Feng et al., 2022).

Considerable inter-subject variability in the plasma CBD concentrations and hence the PK parameters are noted following single oral administration of sesame oil-based CBD formulations (Zgair et al., 2016; Zgair et al., 2017; Feng et al., 2021a; Feng et al., 2021b). Co-administration with high-fat meals also resulted in reduced inter-subject variability compared with fasting state (Perucca and Bialer, 2020). Consistent with the literature, we observed lower inter-subject variability of key PK parameters including the AUC_last_, C_max_ and T_max_ following repeated dosing compared to single dosing. Repeated oral dosing also resulted in a shorter T_max_ especially at higher CBD dose (Taylor et al., 2018; Child and Tallon, 2022; Xia et al., 2025). Based on the understanding of the impact of feeding and lipid composition of the formulations on the oral bioavailability, the repeated oral dosing with lipid rich formulation potentially impacted the physiological state of the animal such as its gastric and intestinal luminal contents, lipid transporter activity and/or gut blood flow, which overall improved the emulsification and absorption of CBD (Wall et al., 1976; Fabritius et al., 2012; Daublain et al., 2017; Feng et al., 2022).

A limitation of the present study is that the *in vivo* PK evaluation was conducted using only one representative synthesized CBD API and was therefore not intended to establish comparative PK equivalence across all four CBD sources. The head-to-head comparison was restricted to the *in vitro* toxicology endpoints. Selection of API2 for PK was based on its GMP-grade status, high purity, and the absence of meaningful API-dependent differences across the *in vitro* assays. Consequently, the PK data should be interpreted as providing exposure and accumulation context for repeated oral administration of a representative purified CBD API, rather than as evidence that all tested CBD sources would necessarily exhibit identical PK behavior *in vivo*.

Systemic accumulation of CBD and the main metabolites upon repeated dosing were observed in the *in vivo* part of this study. The tested CBD dose of 26.5 mg/kg is equivalent to 4.27 mg/kg human dose (extrapolating based on body surface area with a factor of 6.2) or 214 mg/day for a 50 kg subject. In the *in vivo* PK study, repeated dosing resulted in the accumulation of CBD and its primary metabolites. The calculated fold increase in C_max__D was 2.5 (Supplementary Table 6). Although the percentage extrapolation for AUC_INF_ exceeds 15% (at 20.8% in Table 3) relative to AUC_last_ on Day 21, which implies AUC_last_ values being slightly underestimated, this has minimal impact on the calculated fold accumulation in AUC as reflected by the closely similar fold increase of AUC_last__D (i.e. 2.9) and AUC_inf__D (i.e. 3.1) (Supplementary Table 6) upon repeated dosing. The literature has reported mixed findings, likely due to variations in group sizes and dosing regimens. In a reported 90-day oral toxicology study, both C_max_ and AUC_last_ increased following repeated administration of CBD at doses of 5, 15, and 150 mg/kg in male and female rats (Xia et al., 2025). In a separate study with rats receiving 115 mg/kg of CBD for 28 days, an increase in AUC_last_ was observed only in females (Child and Tallon, 2022). In contrast, no significant increases in CBD C_max_ or AUC_last_ were observed after 14 days of repeated inhalation exposure (Schwotzer et al., 2023). The 2.9 to 3.1-fold accumulation of CBD (Supplementary Table 6) observed in this study in male rats was closely similar to that in humans (1.8- and 2.6-fold after 750 and 1,500 mg/day) following 7-day repeated twice per day dosing (Taylor et al., 2018) for CBD. However, the accumulation of 7-COOH-CBD was more prominent (4.5 to 9.6-fold) in humans compared to only about 2-fold accumulation in the rats, potentially linked to the higher 7-COOH-CBD to CBD ratios of the C_max_ and AUC_last_ (30 and 47, respectively at day 7) in humans compared to 2.5 and 2.3, respectively at Day 21 in the rats. Trough CBD plasma levels reached a maximum concentration within about 6 days in this study upon once daily dosing. This was longer as compared to 2 days upon twice-daily dosing in humans (Taylor et al., 2018), potentially due to shorter dosing interval (12 h vs. 24 h) in the 7-day human PK study as well as interspecies differences. CBD exhibits multi-phasic elimination phase and is characterized by long terminal elimination half-life in humans (Taylor et al., 2018). We observed a long terminal elimination phase of 41.9 h and lower K_e_ (0.017 h-1) upon repeated dosing, which contrasted with the unchanged K_e_ values reported in an oral repeated dosing study in rats (Child and Tallon, 2022). The differences between the 2 rat studies could possibly be due to short (24-h) post-exposure phase in Child and Tallon’s study which may miss the later slow clearance phase of CBD when CBD was slowly released from storage sites.

CBD accumulates to different extents in various tissues. Sesame oil promoted intestinal lymphatic transport (Zgair et al., 2016; Feng et al., 2021a; Feng et al., 2021b) and distribution at high levels to the mesenteric lymph nodes at 1.5–2.5-h following oral dosing (Feng et al., 2021a; Feng et al., 2021b). Adipose tissue tends to have the highest CBD levels, followed by liver and muscle (Child and Tallon, 2022; Dehner et al., 2025). Our study has also consistently demonstrated highest CBD accumulation in adipose tissue followed by the MLN and the liver but no accumulation in the brain. While the exact tissue concentrations and fold-accumulation of CBD in various tissues could differ between studies, potentially due to differences in fasting states, older rats and larger fat depot mass in heavier rats, slower release of CBD from adipose tissue compared to other tissues, and different time points when tissues were harvested following dosing. Brain distribution studies indicated that CBD reaches peak concentrations in the brain within 1–2 h post-oral/IP administration, with specific formulations potentially enhancing CBD’s brain bioavailability (Brookes et al., 2023; Binova et al., 2025; Feng et al., 2025). The reported brain-to-plasma ratios differed and ranged either <1 (Deiana et al., 2012) or >1 (Brookes et al., 2023), suggesting the influence of formulations in permeability and accumulation in the brain. The brain-to-plasma ratio in our study, obtained from a single time point (72-h), suggested no accumulation of CBD in the brain during the clearance phase of the repeated dosing study. Further work will need to be performed to assess the tissue PK and tissue-to-plasma ratios at steady state condition. Due to their more polar nature, both 7-COOH-CBD and 7-OH-CBD were much less abundant in the adipose tissue and MLN compared to CBD and were not detected in the brain, liver and lung. In addition to the above-described factors, understanding how feeding status and repeated dosing affect CBD pharmacokinetics is essential for improving the effectiveness of CBD-based treatments.

## 5 Conclusion

Despite differences in origin and purity, the four (−)-*trans*-CBD APIs did not yield any significantly different results in our *in vitro* studies. All samples showed a consistent safety profile, characterized by no genotoxicity and no mutagenicity in standard assays and a very similar *in vitro* hepatotoxicity pattern. These findings suggest that the intrinsic biological effects of CBD overshadow any minor compositional differences among the samples. In practical terms, synthetized and plant-derived CBD (whether high-purity isolate or less-refined extract) appear to have equivalent toxicological behavior *in vitro*. This equivalence is an important insight for regulatory science and risk assessment, indicating that (−)-*trans*-CBD from diverse sources can be considered toxicologically similar regarding genotoxic risk and hepatotoxic potential. Further investigations (e.g., in more complex liver models or *in vivo*) are recommended to confirm these results and to elucidate the mechanisms of CBD-induced hepatotoxicity observed at high doses.

When compared with published data, the *in vivo* PK bioavailability in terms of C_max_ and AUC_last_ was similar between synthetized and hemp-derived CBD isolate. In rats, repeated oral dosing of highly purified (−)-*trans*-CBD led to greater plasma accumulation of CBD and its metabolites compared to single dosing, with particularly notable tissue accumulation of CBD and 7-OH-CBD in the adipose tissue and MLN. These findings demonstrate distinct accumulation patterns and support the conclusion that repeated oral administration significantly affects the pharmacokinetics and tissue distribution of CBD and its main metabolites. These findings will be important to inform on upcoming oral dosing regimens in preclinical research on the CBD pharmacology and efficacy and provide data for the extrapolation of CBD pharmacokinetics in humans.

## Data Availability

The original contributions presented in the study are included in the article/Supplementary Material, further inquiries can be directed to the corresponding author.
