# Supplementary material for: Comparative in vitro safety assessments of synthetized and plant-derived cannabidiol (CBD), and pharmacokinetics of synthetized CBD: evidence of similar toxicological profiles and accumulation upon repeated oral administration
Source: Front Toxicol. 2026 Sep 4;8:1844643. doi: 10.3389/ftox.2026.1844643 (PMC13586287; doi:10.3389/ftox.2026.1844643)
Supplement: Supplementary file 1 [file Supplementaryfile1.docx]

Supplementary Material

# 1 Supplementary Figures and Tables

## 1.1 Supplementary Figures

**Supplementary Figure 1.** Comparisons of plasma CBD PK parameters between synthesized CBD (API 2) and hemp-derived CBD (API 3). Data are presented as mean values of the dose normalized AUC_last_ and C_max_ data. The PK parameters of CBD between current (using synthesized CBD, API2) and published ((Xia et al. 2025) hemp-derived CBD using API3) data are shown for dose normalized area under the curve (AUC_last__D) and maximum plasma concentration (C_max__D).

## 1.2 Supplementary Tables

|  |  |  | **Measured CBD concentration** | | | |
| --- | --- | --- | --- | --- | --- | --- |
| **Name of formulations** | **Theoretical CBD Concentration (mg/mL)** | **Replicate #** | **Measured (mg/mL)** | **Average (mg/mL)** | **%Accuracy** | **% RSD** |
| Synthesized CBD (API2) | 8.857 | 1 | 8.834 | 8.823 | 99.62 | 0.14 |
|  |  | 2 | 8.809 |  |  |  |
|  |  | 3 | 8.825 |  |  |  |
| Synthesized CBD (API2) | 8.857 | 1 | 8.785 | 8.838 | 99.79 | 0.53 |
|  |  | 2 | 8.854 |  |  |  |
|  |  | 3 | 8.874 |  |  |  |

**Supplementary Table 1.** Measured concentrations of CBD oral formulation

Reference

Xia, W., R. Yanuar, V. Mandal, K. Renggli, J. Ho, B. Phillips, G. N. Nikolajsen, S. S. Jensen, H. Z. Bruun, and J. Hoeng. 2025. 'A 13-week oral toxicity and toxicokinetic study of cannabidiol in Sprague Dawley rats with a 4-week recovery period', Drug and Chemical Toxicology: 1-14.

| **Dosing day** | **Average body weight (kg)** | **Achieved delivered dose (mg/kg/day)** | **% Difference from target dose** |
| --- | --- | --- | --- |
| 1 | 0.383 | 26.469 | -0.4% |
| 21 | 0.428 | 26.514 | -0.2% |

**Supplementary Table 2.** Delivered CBD doses in animals (target 26.572 mg/kg) following oral dosing of API2

|  | **Day** | | | | | | | |
| --- | --- | --- | --- | --- | --- | --- | --- | --- |
| Animal ID | **1** | **3** | **7** | **10** | **14** | **17** | **21** | **24** |
| 1001001 | 379.3 | 368 | 384.4 | 394.8 | 406.2 | 409.5 | 425.1 | 412.6 |
| 1001002 | 389.3 | 370.8 | 387 | 394.8 | 409.6 | 419.8 | 438.1 | 426.7 |
| 1001003 | 401.5 | 386.6 | 406.3 | 416.1 | 424.9 | 430 | 443.6 | 437.9 |
| 1001004 | 378.9 | 363.9 | 380.3 | 389.4 | 403.1 | 406.4 | 418.9 | 410.3 |
| 1001005 | 394.7 | 391.9 | 406.9 | 422.5 | 431.9 | 445.8 | 460.1 | 454 |
| 1001006 | 365.4 | 357.4 | 368.5 | 383 | 389.9 | 395.6 | 408.3 | 400.3 |
| 1001007 | 382.5 | 369.2 | 380.4 | 388.7 | 400.1 | 401.1 | 416.4 | 404.9 |
| 1001008 | 367.1 | 361.7 | 367.1 | 378.5 | 382.8 | 386.2 | 389.2 | 380.9 |
| 1001009 | 403.8 | 387.6 | 409.2 | 426.4 | 418.5 | 446.2 | 462.1 | 457.5 |
| 1001010 | 365.2 | 361.6 | 373.2 | 382.4 | 369.7 | 396.3 | 406.5 | 401.6 |
| 1001011 | 381 | 367 | 381.9 | 395.2 | 413.8 | 420.6 | 433.8 | 428.6 |
| 1001012 | 389.2 | 379.7 | 392.9 | 403.6 | 415.4 | 422.6 | 433.7 | 424.9 |
| 1001013 | 376.2 | 363.2 | 381.1 | 387.2 | 401.6 | 405.3 | 423 | 413.9 |
| **N** | 13 | 13 | 13 | 13 | 13 | 13 | 13 | 13 |
| **mean** | **382.6** | **371.4** | **386.1** | **397.1** | **405.2** | **414.3** | **427.6** | **419.5** |
| **SD** | 12.7 | 11.3 | 14.1 | 15.6 | 17.1 | 18.7 | 20.8 | 21.8 |
| **SEM** | 3.5 | 3.1 | 3.9 | 4.3 | 4.7 | 5.2 | 5.8 | 6.0 |
| **RSD (%)** | 3.3 | 3.0 | 3.6 | 3.9 | 4.2 | 4.5 | 4.9 | 5.2 |
| **% of initial mean** | 100 | 97 | 101 | 104 | 106 | 108 | 112 | 110 |

**Supplementary Table** **3.** Body weight progression during and following repeated oral dosing of API2

|  | **AUC_last__D** | **AUC_INF__D** | **C_max__D** | **T_max_** | **T_1/2_** |
| --- | --- | --- | --- | --- | --- |
| Analyte/ Day | h*kg*ng/mL/mg | h*kg*ng/mL/mg | kg*ng/mL/mg | h | h |
| **CBD** | | | | | |
| **Day 1** | 175 ± 56 | 209 ± 46 | 22 ± 12 | 5.5 ± 2.0 | 5.9 ± 2.6 |
| **Day 21** | 446 ± 80 | 567 ± 124 | 46 ± 19 | 4.0 ± 0.0 | 41.9 ± 9.7 |
| **Day 21: Day 1** | 2.9 *** | 3.1 *** | 2.5 *** | 0.8 | 8.3 *** |
| **7-COOH-CBD** | | | | | |
| **Day 1** | 581 ± 179 | NC | 53 ± 15 | 8.8 ± 2.8 | NC |
| **Day 21** | 1006 ± 301 | NC | 106 ± 26 | 8.0 ± 0.0 | 9.6 ± 1.5 |
| **Day 21: Day 1** | 2.0 ** | 1009 ± 302 | 2.2 *** | 1.0 | - |
| **7-COOH-CBD: CBD Day 1** | 3.4 | - | 2.8 | 1.7 | - |
| **7-COOH-CBD: CBD Day 21** | 2.3 | 1.9 | 2.5 | 2.0 | 0.2 |
| **7-OH-CBD** | | | | | |
| **Day 1** | 31 ± 9 | NC | 3.7 ± 1.0 | 8.0 ± 0.0 | NC |
| **Day 21** | 43 ± 4 | 46 ± 4 | 4.1 ± 0.6 | 6.2 ± 2.1 | 26.5 ± 5.3 |
| **Day 21: Day 1** | 1.5 *** | - | 1.2 | 0.8 | - |
| **7-OH-CBD: CBD Day 1** | 0.19 | - | 0.21 | 1.6 | - |
| **7-OH-CBD: CBD Day 21** | 0.10 | 0.08 | 0.10 | 1.5 | 0.68 |
| **6-OH-CBD** | | | | | |
| **Day 1** | 13 ± 5 | NC | 1.5 ± 0.4 | 8.0 ± 0.0 | NC |
| **Day 21** | 33 ± 3 | 37 ± 3 | 3.7 ± 0.6 | 8.0 ± 0.0 | 57.0 ± 33.2 |
| **Day 21: Day 1** | 2.8 *** | - | 2.7 *** | 1.0 | - |
| **6-OH-CBD: CBD Day 1** | 0.08 | - | 0.09 | 1.6 | - |
| **6-OH-CBD: CBD Day 21** | 0.08 | 0.07 | 0.10 | 2.0 | 1.5 |

**Supplementary Table 4.** Pharmacokinetics parameters of CBD, 7-COOH-CBD, 7-OH-CBD and 6-OH CBD following oral dosing of API2. Data shown are mean and standard deviation of the PK parameters for dose-normalized area under the curve (AUC_last__D and AUC_inf__D), maximum plasma concentration (C_max__D), time to maximum plasma concentration (T_max_) and half-life (T_1/2_) for CBD, 6-COOH-CBD, 7-OH-CBD and 6-OH-CBD. The average ratios of the respective parameters obtained on day 21 versus day 1 as well as metabolites to CBD ratios are indicated. N =8-13 male rats. NC, not calculable. *, **, and *** refer to statistical significance at p<0.05, p<0.01, and p<0.001, respectively, at day 21 versus day 1.

| **Tissue to plasma ratio** | **Brain** | **Liver** | **Lung** | **Adipose** | **MLN** |
| --- | --- | --- | --- | --- | --- |
| CBD | 0.3 ± 0.1 (7) | 1.5 ± 0.4 (13) | 0.7 ± 0.6 (10) | 1470.0 ± 1047.0 (13) | 201.3 ± 83.5 (13) |
| 7-COOH-CBD | NC | NC | NC | 10.0 ± 7.4 (9) | NC |
| 7-OH-CBD | NC | NC | NC | 20.9 ± 11.4 (8) | 73.4 ± 29.3 (13) |
| 6-OH-CBD | NC | NC | NC | NC | NC |

**Supplementary Table 5.** Tissue to plasma concentration ratios of CBD and its metabolites following oral dosing of API2. The tissue to plasma ratios for CBD and its metabolites are presented as mean and standard deviation with the number of independent subjects in parenthesis. The tissue to plasma concentration ratio is indicated NC when the concentration in the tissues were <LOQ. MLN, mesenteric lymph nodes; NC, not calculable.
